# The Efficacy and Harms of Pharmacological Interventions for Aggression After Traumatic Brain Injury—Systematic Review

**DOI:** 10.3389/fneur.2019.01169

**Published:** 2019-11-29

**Authors:** Amelia J. Hicks, Fiona J. Clay, Malcolm Hopwood, Amelia C. James, Mahesh Jayaram, Luke A. Perry, Rachel Batty, Jennie L. Ponsford

**Affiliations:** ^1^Monash-Epworth Rehabilitation Research Centre, Turner Institute for Brain and Mental Health, Monash University, Melbourne, VIC, Australia; ^2^Department of Psychiatry, University of Melbourne, Melbourne, VIC, Australia; ^3^Department of Forensic Medicine, Monash University, Southbank, VIC, Australia; ^4^Professorial Psychiatry Unit Albert Road Clinic, Department of Psychiatry, University of Melbourne, Melbourne, VIC, Australia

**Keywords:** traumatic brain injury, TBI, aggression, irritability, pharmacotherapy, intervention, review

## Abstract

**Background:** Aggression is a commonly reported problem following traumatic brain injury (TBI). It may present as verbal insults or outbursts, physical assaults, and/or property destruction. Aggressive behavior can fracture relationships and impede participation in treatment as well as a broad range of vocational and social activities, thereby reducing the individual's quality of life. Pharmacological intervention is frequently used to control aggression following TBI. The aim of this systematic review was to critically evaluate the evidence regarding efficacy of pharmacological interventions for aggression following TBI in adults.

**Methods:** We reviewed studies in English, available before December 2018. MEDLINE, PubMed, CINAHL, EMBASE, PsycINFO, and CENTRAL databases were searched, with additional searching of key journals, clinical trials registries, and international drug regulators. The primary outcomes of interest were reduction in the severity of aggression and occurrence of harms. The secondary outcomes of interest were changes in quality of life, participation, psychological health (e.g., depression, anxiety), and cognitive function. Evidence quality was assessed using the Cochrane Risk of Bias tool and the Joanna Briggs Institute Critical Appraisal Instruments.

**Results:** Ten studies were identified, including five randomized controlled trials (RCTs) and five case series. There were positive, albeit mixed, findings for the RCTs examining the use of amantadine in reducing irritability (*n* = 2) and aggression (*n* = 2). There were some positive findings favoring methylphenidate in reducing anger (*n* = 1). The evidence for propranolol was weak (*n* = 1). Individual analysis revealed differential drug response across individuals for both methylphenidate and propranolol. The less rigorous studies administered carbamazepine (*n* = 2), valproic acid (*n* = 1), quetiapine (*n* = 1), and sertraline (*n* = 1), and all reported reductions in aggression. However, given the lack of a control group, it is difficult to discern treatment effects from natural change over time.

**Conclusions:** This review concludes that a recommendation for use of amantadine to treat aggression and irritability in adults following TBI is appropriate. However, there is a need for further well-designed, adequately powered and controlled studies of pharmacological interventions for aggression following TBI.

## Introduction

### Rationale

Problems with aggression, including agitated and irritable behavior, anger, verbal outbursts, physical assaults and property destruction (1–6), are common after traumatic brain injury (TBI) (7). Indeed, a recent review of epidemiological studies found an incidence of verbal and physical aggression post TBI across the spectrum of severity of 25–39% (8). The evolution and resolution of symptoms over time varies greatly (6), with aggression persisting for many decades after injury in some individuals (1–4, 9, 10). Aggressive behavior may limit access to rehabilitative treatment, participation in employment and in valued community activities (e.g., sports clubs, volunteering), as well as contribute to loss of friendships and romantic relationships (3, 4, 9, 11, 12). At the more severe end of the spectrum, aggressive behavior post TBI can result in violent crime, intimate partner violence, and ultimately incarceration (13–15).There may be unwanted changes in important family roles (3) due to strained marital relationships and difficulties with caring appropriately for children (3, 16). Family members have reported pervasive fear of aggressive outbursts, with concerns for their physical safety and potential legal consequences for the individual with TBI (5, 11, 17–19). Episodes of verbal and physical aggression can be traumatizing for families, leading to depression and anxiety in family members (3, 5, 11, 17–19). Aggression may also increase the burden of caring for the person with TBI, causing financial strain, change in relationships with other family members and result in lower quality of life (3–5, 17, 19).

The etiology of aggression following TBI is complex and multi-faceted (6, 20). Each occurrence of aggressive behavior is thought to be the product of varied interactions between damaged neural systems, cognitive impairments, and pre-morbid factors, which are exacerbated by post-injury and environmental factors. Within the relatively limited neurobiological research to date, damage to the frontal lobes, specifically the pre-frontal cortex, has been consistently associated with aggression post TBI (21–23). Pre-morbid factors may include personality traits, poor social functioning, pre-existing mental illness, and substance abuse (20, 22). Post-injury factors include medical and psychiatric comorbidities, poor emotional insight, medication, problems with sleep and fatigue, and environmental factors including interactions with family/carers, financial strain, and lack of control over everyday activities and being placed in overly demanding situations (3, 4, 6, 7, 9, 11, 20, 22, 24–27). With respect to comorbid psychiatric conditions, depression is thought to be strongly associated with aggression (7, 8, 12, 22, 28). In the acute stages of recovery from TBI when the patient is in post-traumatic amnesia (PTA), aggression appears to be largely underpinned by confusion, disorientation, and generalized cognitive impairments that resolve to a significant degree with emergence from this state. Aggression does not extend beyond PTA for many patients (29). Therefore, it is arguable that clinicians should differentiate between aggression occurring in the post-acute period, and agitation within the period of PTA. This review will therefore focus on post-acute management of aggression.

Numerous options for the management of aggressive behavior post TBI have been advocated in the literature, including both pharmacological and non-pharmacological approaches (30). Non-pharmacological treatment methods, including cognitive behavioral therapies, psychotherapy, relaxation-based therapies, skills-training programs, exposure-based treatments, behavioral interventions, and multicomponent treatments, have shown varying levels of success (31–33). Pharmacological methods are more commonly used. Given there are no FDA (Food and Drug Administration) approved medications for aggression post TBI, all medication is prescribed off-label (34, 35). As such, clinicians must rely on their clinical expertise, experience in treating similar conditions, extrapolation of aggression management from non-TBI populations, and consideration of other factors that may preclude certain medications such as availability and cost (34, 36). This has facilitated wide variation in prescribing practices, with anti-convulsants, anti-depressants, and anti-psychotics being the most commonly prescribed (35, 37).

Although this topic has attracted a number of previous reviews, many are outdated and their conclusions are limited by methodological issues, for example, lack of key systematic review components (no comprehensive search for published and unpublished data; lack of comprehensive evidence tables; no methodological assessment for risk of bias) (36, 38–43), failure to examine harms (40), and absence of a clear delineation between studies in which participants were in or out of PTA (34, 36, 44, 45). With respect to the findings from previous reviews, all have agreed that no strong conclusions could be drawn due to the limited number of studies and overall weakness of the evidence for each class of medication (34, 39, 41). Notwithstanding this, there was a general consensus that the current best evidence for treatment of aggression post TBI supports the use of amantadine and beta-blockers, with typical neuroleptics only to be prescribed with caution due to concern regarding adverse events (34, 36, 38, 41, 42, 44–47). Many other drugs have also been listed as possible options including anticonvulsants (mostly carbamazepine, valproic acid), specific serotonin reuptake inhibitors (SSRIs), tricyclic antidepressants (TCA), atypical antipsychotics, methylphenidate, and lithium (40–42, 44, 46, 47).

Due to the lack of robust clinical research and strong recommendations from reviews, guidelines for the management of post-TBI aggression are largely absent. Two identified guidelines, which are now between 6 and 13 years old, provide some recommendations, albeit limited by the poor quality of the studies and reviews on which they were based (48, 49). Both guidelines advocated for beta-blockers (propranolol and pindolol) as a treatment option, with the earlier guideline from 2006 also listing methylphenidate, SSRIs, valproate, lithium, TCAs, and busiprone as alternative treatment options (48).

### Objectives

In light of the paucity of evidence regarding management of aggression post TBI for patients who have cleared PTA, the present study aimed to systematically review the efficacy and harms of pharmacological therapies, as compared to all types of comparators, for aggression post TBI. This review also examined, as secondary outcomes, quality of life, participation, changes in psychological health (e.g., depression, anxiety, distress), and cognitive function. This review specifically addresses limitations in the extant literature by including a rigorous and comprehensive literature search, examination of harms, and methodological assessment for risk of bias. Further, only studies of post-PTA samples are included to ensure that the review focuses only on aggression after emergence from PTA. This provides clinicians with a thorough and detailed examination of all relevant evidence upon which to base prescribing decisions.

### Research Question

The specific review question was: What are the efficacy and harms of pharmacotherapy as compared to all other comparators for the management of aggression in adults 16 years and over who have sustained a TBI?

## Methods

To ensure complete and transparent reporting, this review was conducted and reported according to the Preferred Reporting Items for Systematic Review and Meta-Analysis (PRISMA) guidelines (50–52), and the reporting standards for literature searches and report inclusion criteria (53). The protocol for this review was published on the PROSPERO database (54). There were four deviations from the protocol, as described in Table 1.

**Table 1:** Deviations from protocol.

**Original criteria**	**Change**	**Justification**
Eligible participants must be adults aged 18 years and above	Participants were required to be adults aged 16 years and over, of either gender (studies where >80% of the sample was within this age range were also eligible)	The minimum age accepted for inclusion was reduced from 18 to 16 years old in order to be consistent with international definitions of the start of adulthood. The inclusion criteria was also broadened to state that 80% or more of the participants had to be in this age range. This catered for studies in which the inclusion criteria did not include an age range and/or when the sample age was only provided as mean and standard deviation with no range provided
Traumatic brain injury of any severity will be accepted for the review—as diagnosed using any recognized criteria	“Recognized criteria” (i.e., GCS, PTA, LoC, coma) were not required for inclusion	Inclusion criteria broadened due to paucity of studies. Review authors agreed that if the study authors deemed the patient to have had a TBI and/or were assessing them in a hospital/rehabilitation outpatient setting and/or the cause of the injury was clearly TBI (e.g., gunshot wound), this was sufficient
RCTs to be excluded	RCTs were eligible for inclusion in this review	The original intention was to separate this review into two reviews; focusing on RCTs and non-RCTs. However, due to the paucity of studies, it was decided to combine the study designs in a single review
Risk of bias will be assessed using the Newcastle-Ottawa Scale	Risk of bias was assessed using the Cochrane tools (RCTs) and the Joanna Briggs Institute tools (non-RCT)	The review team felt these tools provided a clearer assessment of methodological quality

*GCS, Glasgow Coma Scale; LoC, loss of consciousness; PTA, post-traumatic amnesia; RCT, randomized controlled trial; TBI, traumatic brain injury*.

### Data Sources and Searches

In collaboration with an information specialist, we developed a comprehensive search strategy. Included were terms relating to the population (TBI) and intervention (pharmacotherapy). As this review forms part of a larger project reviewing evidence on a range of neurobehavioral symptoms (NBS) post TBI, aggression was not specified. The search strategy, undertaken on the title and abstract of records, used both keywords and controlled vocabulary with Boolean connectors. To source the keywords, the Cochrane Library and PubMed were searched: specifically, the titles, abstracts, and search strategies of relevant published systematic reviews.

All English language studies, regardless of publication status, available before December 2018, were eligible for inclusion (initial search undertaken in November 2016 and updated in May 2017, November 2017, and November 2018). The following databases were searched by the information specialist: MEDLINE [OVID SP interface (search strategy presented in Appendix I)]; PubMed (excluding MEDLINE); EMBASE (Excerpta Medica Database) (excluding MEDLINE, OVID SP interface); CENTRAL, and two discipline specific databases; PsycINFO (OVID SP interface); CINAHL (Cumulative Index to Nursing and Allied Health Literature).

Supplementary searching by author AH—who has received training in systematic review methodology and conducted previous searches—was undertaken in Research Gate and Google Scholar; international drug regulator websites (Food and Drug Administration, European Medicine Agency and the Medicines and Healthcare Products Regulatory Agency); clinical trial registries (the International Clinical Trials Registry Platform Search Portal and ClinicalTrials.gov; using search terms “traumatic brain injury” and “pharmacotherapy”); hand searching titles in key journals [*Brain Injury* (1987 to March 2019), *Neuropsychology* (1987 to March 2019), *Journal of Neurotrauma* (1988 to March 2019), and *Journal of Head Trauma Rehabilitation* (1986 to March 2019)]; and by contacting academic and clinical experts chosen by the chief investigators (*n* = 10 contacted and responded). The reference lists, citations, and related articles were reviewed for all included studies and any relevant previous reviews of pharmacotherapy for TBI.

### Inclusion Criteria

Studies were selected for this review on the basis of study design, participants, interventions, comparators, and outcomes.

### Types of Studies

The following study types, regardless of sample size and study setting, were considered for inclusion: randomized controlled trials (RCTs), controlled non-randomized clinical trials, quasi-RCTs, controlled before and after studies, interrupted time series with a control group, interrupted time series without a parallel concurrent control group, analytical observational studies (including cohort and case–control studies), case series with pre-test/post-test outcomes, and single arm studies. All studies had to include a baseline measurement, and the aim of the study had to be the treatment of aggression.

### Types of Participants

This review included participants who had sustained a TBI of any cause or severity, and presented with aggression after emergence from PTA. Participants were required to be adults aged 16 years and over, of either gender (studies where >80% of the sample was within this age range were also eligible). TBI had to be defined using recognized criteria such as brain imaging, loss of consciousness, Glasgow Coma Scale (GCS) score, or PTA. Where these were not provided, it was deemed sufficient that the study authors referred to the injury as “TBI” or “head trauma”; the patient was seen in a hospital/outpatient rehabilitation setting, and the cause of the injury was clearly TBI (e.g., gunshot wound to the head). Studies of acquired brain injury populations were only considered if the participants with TBI could be disaggregated. Although there were no restrictions on time since injury, participants had to be clear from PTA. If the sample appeared to contain both participants in PTA and out of PTA at baseline, studies were included if the data could be disaggregated or if >80% of the sample were not in the PTA period at any point during the study.

Aggression was conceptualized as either verbal or physical acts against property, others or self, and included descriptions of “agitation,” “anger,” and “irritability.” Aggression that was sexual in nature was not included. Studies were accepted if they measured aggression using a validated assessment tool {e.g., Overt Aggression Scale-Modified for Neurorehabilitation (OAS-MNR) (55) or the Agitated Behavior Scale (ABS) (56)}. Medical/nursing notes or a log book were accepted if the results were presented quantitatively; qualitative descriptions of the behavior change were not deemed sufficient.

### Types of Interventions

All pharmacotherapy interventions were eligible for inclusion in the review, with no restrictions on dose, duration, frequency, timing of delivery, or combination of drugs. Studies reporting mixed interventions (e.g., pharmacotherapy and psychological therapy) were considered for inclusion, provided the data for the pharmacotherapeutic intervention was reported separately.

### Types of Comparators

All types of comparators were eligible for inclusion, including placebo, standard care, other non-pharmacological therapeutic intervention, and comparison of drugs within the same class. Studies of complementary medicines and over-the-counter medicines were included if they were used as a comparator or a co-intervention to a study drug.

### Types of Outcomes

The primary outcomes of interest for this systematic review were changes in aggression (including changes in severity, frequency or type of aggression) and occurrence of harms. The secondary outcomes of interest were quality of life, participation, changes in psychological health (e.g., depression, anxiety, distress), and cognitive function. Studies were included if they reported on at least one primary outcome.

### Study Selection

Throughout the study selection process, reviewers were not blinded to the journal titles, study authors, or their institutions. Titles and abstracts of all identified publications were screened by two independent reviewers for eligibility (AH and RB; FC and LP), and discrepancies were adjudicated by a third team member. Eligible citations were retrieved in full and assessed by pairs of independent team members (AH and RB; FC and LP), with disagreements resolved through discussion with a third reviewer.

### Data Extraction and Assessment of Methodological Quality

For studies fulfilling inclusion criteria, three authors (AH, FC, and AJ) independently extracted data using a pre-piloted customized data extraction tool based on the standardized tool from the Joanna Briggs Institute System for the Unified Management, Assessment, and Review of Information (JBI-SUMARI) (57). The following data were abstracted: basic study identifying information (author names, publication year, country, financial support received), study methodology (design, sample, population, main inclusion and exclusion criteria, definition and measurement scales used for TBI and aggression, pharmacotherapy, comparator condition, co-intervention, outcomes, statistical analyses), study sample, and findings. All data extracted were checked and verified by the first author (AH). One author was contacted and provided clarification about study characteristics. Data were summarized using tables and narrative synthesis, with results grouped by the primary and secondary outcomes of interest. Study quality was independently assessed by three reviewers (AH, FC, and AJ) using the Joanna Briggs Institute critical appraisal instruments, with all decisions and supporting justifications reviewed and confirmed by author AH (57).

## Results

### Study Selection

**Figure 1:**
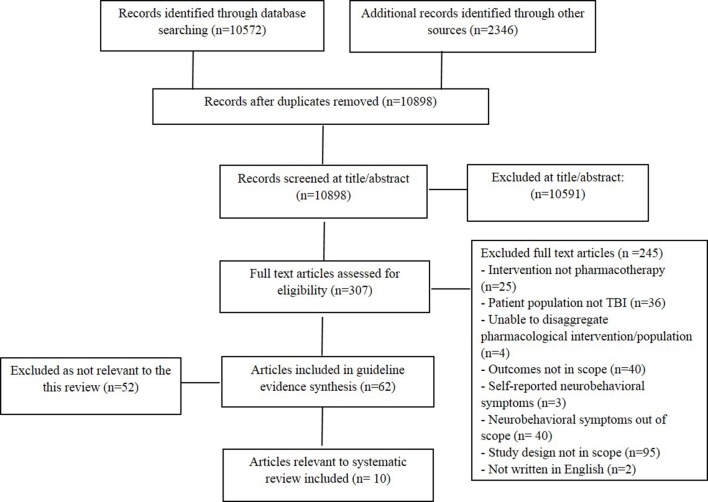
Summary of studies excluded from narrative synthesis.

The literature search produced 12,918 articles, 10,572 from bibliographic databases and 2,346 from additional search sources. Title and abstract screening was completed on 10,898 articles, after 2020 duplicates were removed. Of the 307 articles reviewed at full text, 62 were deemed eligible for inclusion in the broader review (encompassing all NBS post TBI), with 10 studies eligible for the current review. Figure 1 outlines the screening process and reasons for exclusion.

### Study Characteristics

The 10 included studies were published between 1987 and 2017, and comprised 5 RCTs and 5 case series. The majority of studies were published in the USA, with one study from France. The sample sizes varied widely for the RCTs, ranging from 13 to 168, with the case series all having small sample sizes that ranged between 2 and 13 participants. Across the studies, there were more male participants; however, female participants were well-represented within many studies. The majority of participants were aged in their late 30s to early 40s. For those studies that reported on TBI severity, the full spectrum of severity, from mild to very severe, was captured. A variety of pharmacological interventions were examined, including anti-parkinsonian drugs (*n* = 3), anti-epileptics (*n* = 3), neurostimulants (*n* = 1), beta-blockers (*n* = 1), anti-depressants (*n* = 1), and anti-psychotics (*n* = 1).

Both primary outcomes for the review (i.e., changes in aggression and occurrence of harms) were addressed in each of the 10 studies, although, in one study, the findings with respect to harms were not provided (58). Of the secondary outcomes (i.e., quality of life, participation, changes in psychological health, and cognitive function), quality of life and participation were not addressed in any study and as such are not commented on further in this review.

### Randomized Controlled Trials

The five RCTs examined the efficacy of methylphenidate (59), propranolol (60), and amantadine (61–63). Thirty-eight male patients with TBI were administered methylphenidate (up to 30 mg/day) or placebo in a single-blind RCT for 6 weeks. The outcomes examined were changes in aggression and anger measured on four validated assessment tools, the occurrence of harms, and changes in psychological health and cognition (59). A double-blind crossover design was used to examine the effects of propranolol (initial dose of 60 mg and up to max 180 mg) on 13 patients more than 1 year post injury on agitation measured on the ABS and the occurrence of harms (60). Hammond et al. conducted all three studies examining the effects of amantadine using a parallel group, randomized, double-blind, placebo-controlled trial (61–63). The 2014 study (61) enrolled 76 patients, an average of 4–5 years post injury, who were administered either amantadine (100 mg; 2/day) or placebo for 28 days. Neuropsychiatric Inventory (NPI) subscales were used to examine irritability, agitation, and aggression. The occurrence of harms and impacts to psychological health was also measured. A large sample of 168 patients was included in the 2015 study (63), of whom 86 received placebo and 82 patients were administered amantadine (100 mg; 2/day) for 60 days. The irritability subscale of the NPI was used, along with a measure of harms and psychological health. The 2017 study (62) examined a subset of 118 individuals from the 2015 study (63) who had moderate to severe aggression, to examine the impact of amantadine on NPI agitation and aggression subscales, state and trait anger scores, as well as anger expression in this specific group.

### Case Series

Five studies used an open label case series design to examine the effects of carbamazepine (64, 65), valproic acid (66), sertraline (58), and quetiapine (67) on aggression post TBI. Using a prospective open label trial, Azouvi et al. (64) examined the impact of carbamazepine (initial dose of 200 mg and up to max 800 mg) on agitation and anger outbursts in 10 patients over an 8 week period. The occurrence of harms and changes in cognitive function was also measured. The impact of carbamazepine was also examined in a second study, from which data on two patients with TBI could be extracted (65). The drug was administered over a 2 week period (increased from an initial dose of 200 mg 3/day until carbamazepine level could be obtained−8–12 μg/ml), with changes in assaultive behaviors and occurrence of harms documented. Similarly, the data for two TBI patients were extracted from a study by Wroblewski et al. (66) that also examined another anti-convulsant medication, valproic acid. Patient 1 was approximately 5 years post his injury and was administered 750 mg of valproic acid per day for 3 months. Patient 2 was 2.5 years post injury and was administered an initial dose of 500 mg per day of valproic acid, which was subsequently titrated up to a maximum dose of 1,500 mg per day over the 6 week data collection period. The impact of these interventions was documented by counts of acts of physical aggression and “time outs” for verbal aggression, as well as the occurrence of harms. Sertraline was administered to a group of 13 mostly male patients over an 8 week period. The initial dose was 50 mg per day, and this was titrated to 200 mg per day or the maximum tolerable dose. Irritability and aggression scales of the outpatient Overt Aggression Scale (OAS) were used, along with examination of harms and psychological health. Seven patients an average 1 year post injury were administered quetiapine over a 6 week period (initial dose 50–100 mg per day; maximum dose ranged from 25 to 300 mg) to examine the effects on the OAS, aggression subscale of the Neurobehavioral Functioning Inventory and clinician impression of change. Harms and impact on cognition were also assessed.

### Synthesized Findings

#### Changes in Aggression/Anger/Irritability/Agitation

##### RCTs

Overall, the findings for the efficacy of methylphenidate, propranolol, and amantadine for treating post-TBI aggression were mixed, with analysis suggesting differential response patterns across individuals (59–63).

The efficacy of methylphenidate was assessed across four separate outcome measures, as well as an overall combined analysis across measures using hierarchical clustering (59). Methylphenidate was associated with a significant reduction in scores for trait anger, state anger, hostility, and belligerence (59). Hierarchical clustering produced two clusters within the treatment group; the “non-responders” (no reduction in anger scores from baseline to 6 weeks) and the “response group” (all members exhibited clear reduction in anger from baseline to 6 weeks). Discriminant analysis revealed that participants with higher baseline anger scores were more likely to respond to the drug than participants with low baseline anger scores (59).

The findings for efficacy of propranolol were mixed. Across the 10 patients, the magnitude of change in behavior measured by the ABS from baseline to intervention phase was 0.135, which denotes a “small or negligible” change. Individual analysis revealed three groups of response type; little or no effect (*n* = 6), moderate to strong effect—improvement (*n* = 2), and moderate to strong effect—worsening (*n* = 2).

The three studies of amantadine examined irritability, aggression, and anger (61–63). The findings with respect to irritability differed between studies. In the 2014 study, there was a greater reduction in overall irritability, as well as in the frequency and severity of the most problematic irritable behavior, over the 28 day intervention in the treatment group from the perspective of the observer (61). However, there was no significant change in the distress associated with the behavior (61). In comparison, the 2015 study examining amantadine use over a 60 day period found no significant differences between the groups (either at 28 or at 60 day follow-up) for the most problematic irritability behavior, most aberrant behavior, or the distress associated with the irritable behavior (from either the perspective of the participant or the observer) (63).

With respect to change scores for aggressive behaviors and associated distress, there was no significant difference in the change scores for the treatment and placebo groups from the perspective of an informant (61, 62). However, when the sample was restricted to only those who had scored >2 on the NPI-A, a significant difference in change scores was noted (61). Further, when participant ratings were collected in the 2017 study, there was statistically significant difference between the groups regarding the change in aggression scores and distress related to the behavior for Day 60, although not Day 28 (62). The 2017 study also examined state and trait anger as well as anger expression, finding no significant differences in group change scores (62).

##### Case series

The cases series provided mostly positive findings. Both the case series examining the effects of carbamazepine reported positive findings overall, with a reduction in the number of assaultive behaviors (65) and a significant improvement in irritability and disinhibition on the NRS-R and the ABS (64). Conversely, there was no significant change in hyperactivity–agitation, mood lability, excitation, or hostility on the NRS-R (64). There was also inter-individual variability in treatment response on the NRS-R; five patients showed a decrease over the intervention of 50% or more, three patients' scores decreased between 25 and 43%, and two patients showed no change (64). The two patients administered valproic acid showed improvements in verbal abuse, yelling, threats of assault, and physical aggression (66). Significant improvements were reported for both aggression and irritability for patients treated with sertraline, with 80% and 100% of patients demonstrating a clinically meaningful improvement at 8 weeks for aggression and irritability, respectively (58). For those administered quetiapine, there was a significant reduction in aggression documented on both the aggression subscale of the NFI and the OAS-M, and an improvement on the Clinical Global Impression scale (67).

#### Harms

Only one study did not report on the occurrence of harms (although it did state that clinical assessment for harms took place at each follow-up visit) (58). No adverse events were reported for valproic acid (66) or methylphenidate (59). Adverse events were reported for administration of carbamazepine (64), quetiapine (67), and propranolol (60). With respect to amantadine, there were no significant group differences in proportion or severity of adverse events (61–63); however, one participant required drug termination secondary to a seizure (61). Carbamazepine was associated with drowsiness (*n* = 4), requiring lowering of dosage, and a single serious adverse event was recorded, in which a patient experienced a significant allergic cutaneous reaction toward the end of the intervention period (day 51 of 56), requiring withdrawal of medication (64). Transient diplopia and ataxia, clearing spontaneously within 1 h were also reported (65); however, it was unclear if this occurred in the two TBI patients included in the current review. Quetiapine was associated with mild extrapyramidal side effects and akathisia in one patient, and three patients reported sedation that resolved by weeks 3 to 6 (67). Propranolol administration resulted in a paradoxical increase in agitation for two patients (60).

#### Cognition

There was no impact on cognitive functioning reported for carbamazepine (64) or methylphenidate (59). In contrast, there was a significant improvement in cognitive functioning on the RBANS (Repeatable Battery for the Assessment of Neuropsychological Status) for those administered quetiapine (67).

#### Psychological Health

Four studies addressed the impact of the intervention on psychological health. The administration of sertraline was associated with an improvement in depression scores at the 4 week follow-up, but not at the 8 week follow-up (58). There was no impact of sertraline on suicidality at either follow-up (58). There was an overall improvement in general psychopathology for those administered methylphenidate, as well as significantly greater reductions in the presence and severity of brain-injury related personality changes, as rated by both the patient and an informant (59). Amantadine was not associated with changes in scores on global mental health, depression, or anxiety symptoms, as rated by the participant or observer (61, 63). Conversely, the clinician rated Clinical Global Impressions–Global Improvement subscale did show greater global improvement for the treatment group at 60 day follow-up, but not at the earlier 28 day follow-up (63).

#### Other

Overall behavior was also noted to improve with carbamazepine, as was social functioning (64).

#### Risk of Bias

The five RCTs were assessed as having low to moderate risk of bias. Most commonly, studies did not clearly report how the random sequence was generated, how allocation concealment was maintained, or what were the procedures for blinding of participants, personnel, and outcome assessors. The case series were judged to have moderate to high risk of bias, and were inherently limited by lack of a control group. The areas of methodological weakness varied across studies and are outlined in Supplementary Materials. Across studies, there was some detail provided regarding co-interventions (e.g., drug class and a statement that dosage was stable during intervention). Three studies provided no information regarding co-interventions (58–60).

## Discussion

### Summary of Main Findings

The primary aim of this systematic review was to evaluate the evidence for efficacy and harms of pharmacological interventions for aggression following TBI. Ten studies met inclusion criteria: five RCTs and five case series. Multiple studies examined the effects of anti-parkinsonian and anti-epileptic medications, with the remaining studies using neurostimulants, beta-blockers, anti-depressants, and anti-psychotics. Overall, this review concludes based on the evidence from three RCTs conducted in an outpatient community-based setting that there is sufficient evidence to make a recommendation for the use of amantadine in treating aggression and irritability after TBI in the post-PTA period.

The primary outcome, change in aggressive behavior, was measured in all studies included in the review. Three RCTs examined the impact of amantadine on irritability, aggression, and anger (61–63). It is postulated that amantadine may improve irritability and aggression through enhancing cognitive function and, through this mechanism, may enhance cognitive appraisal and behavioral disinhibition (61, 68). Overall, there was some positive, albeit mixed, findings for an effect of amantadine on irritability and aggression in community-based samples (61–63), with no evidence found for reducing anger (62). The impact of amantadine on irritability was examined in two studies (61, 63). Both studies used a 28-day follow-up time point, with a further 60 day follow-up also included in the 2015 study. At the 28 day follow-up, only one of the two studies found a significantly greater reduction in irritability in the treatment group (61). However, when the results from these studies were combined in a meta-analysis, the pooled result did favor amantadine over placebo (63). At the 60 day follow-up, included in the 2015 study, there was no significant impact of amantadine on irritability (63).

The impact of amantadine on aggressive behavior was measured in two RCTs (61, 62). There was some evidence in favor of amantadine in treating aggression; however, outcomes varied for different follow-up time points, respondent types (i.e., participants vs. informants), and baseline aggression levels across the samples. For example, in the 2014 study, there were no significant differences found in the change scores for the treatment and placebo groups at 28 day follow-up (61). However, when the sample was restricted to only those with more severe aggression (score of >2 on NPI-A), a significant difference in change scores was noted. Although this suggests that amantadine may be more effective for those with more severe aggression, the finding is difficult to interpret in light of the non-significant findings at the 28 day follow-up in the 2017 study that restricted their sample to those with even greater aggression (inclusion criteria of 6 or more on NPI-A). With respect to the possible influence of time point and respondent type, significant results were found in the 2017 study for only the 60 day follow-up, and only from the perspective of the participant. The results for the 28 day follow-up were non-significant for both respondents and non-significant for the informants at the 60 day follow-up. Taken together, these results suggest that participants themselves with more severe aggression may notice an impact of amantadine on aggression over longer time periods.

The mixed evidence reported for amantadine in the Hammond studies may have been contributed to, in part, by a large placebo effect masking detection of a treatment effect (63). Indeed, findings from the control groups showed a reduction in aggression and irritability over the treatment period on a number of the measures administered (61–63). The placebo effect may have resulted from numerous factors including therapeutic alliance, the effect of behavior monitoring, inconsistency in baseline behavior month to month, participant expectations, and other non-specific effects (63). Although the research staff did not interact with patients in an explicitly psychotherapeutic manner, a type of therapeutic alliance may still have formed through kind and supportive interactions (63). It is suggested that larger sample sizes are required to power studies to find treatment effects in the context of robust placebo effects. Finally, the contrasting results from Hammond et al. (61–63) highlight the importance of including the perspectives of the participant, informant, and clinician, and ensuring that the intervention period and time points for follow-ups are of a sufficient duration to allow people to notice a change in behavior (63).

The remaining two RCTs examining the effects of propranolol and methylphenidate demonstrated mixed findings, with further analysis revealing inter-individual differences in response to these medications (59, 60). Specifically, the response to both propranolol and methylphenidate varied across study participants, with some responding favorably to the medication and others not showing any improvement in behavior (59, 60). Further, for 2 of the 10 patients administered propranolol, there was a worsening of behavior (60). Mooney and Haas (59) further analyzed those participants who responded favorably to methylphenidate, identifying that this group had, on average, higher baseline anger scores. Although this may suggest the simple effect of regression to the mean, the authors conducted the same analysis in the control group and found that those with higher anger scores at baseline did not show significantly greater change over time compared to those with lower anger scores at baseline. This suggests that a methylphenidate intervention may be more appropriate for those with more severe aggressive behaviors post TBI, and may be less efficacious for those with milder difficulties.

The case series provided positive findings for use of valproic acid (66), sertraline (58), and quetiapine (67). Of the two studies examining carbamazepine, one reported uniformly positive findings (65), with mixed findings reported by Azouvi et al. (64), who found inter-individual variability in treatment response. Although these findings provide some support for the use of each of these drugs in specific individuals, lack of a control group limits the conclusions that can be drawn, as the studies cannot account for natural recovery over time. Of note, of the two patients administered valproic acid, a drop in serum concentration coincided with a flare in behavior, which resolved with increasing the valproic acid dose (66). This suggests that the change in behavior may have been, at least in part, influenced by administration of valproic acid.

Of the nine studies that reported on harms, no adverse events were reported for valproic acid (66) or methylphenidate (59). Adverse events were reported for carbamazepine (64), quetiapine (67), propranolol (60), and in one of the amantadine studies (61). Carbamazepine was associated with drowsiness, a single significant allergic cutaneous reaction requiring withdrawal of medication (64), and transient diplopia and ataxia (65) (it was unclear whether this occurred in the two TBI patients included in the current review). Quetiapine was associated with mild extrapyramidal side effects and akathisia in one patient, and three patients reported sedation that resolved (67). Propranolol administration resulted in a paradoxical increase in agitation for two patients (60). Although adverse events were mostly non-serious and, in some cases, transient, the impact of harms must be considered in the context of TBI, that is, how much the undesired effect weighs on the neurological recovery of a patient who may also have a cognitive and motor deficit, and the impact of such effects on the patient's ability to engage with other rehabilitation services (69). For example, drowsiness, which may be considered a minor and manageable issue in non-TBI populations, may significantly impact an individual with TBI who is already challenged by significant fatigue and is attempting to engage in demanding cognitive and physical tasks such as physiotherapy. Finally, to increase transparency and consistency among trials, the use of standard reporting of adverse events, for example, using terminology from MEdDRA (Medical Dictionary for Regulatory Activities), is recommended.

Only two of the four secondary outcomes were reported on across the RCTs and case series: cognitive function and psychological health. Only quetiapine was associated with a positive change in cognition (67), with the other two studies reporting on this outcome failing to find a significant impact of carbamazepine (64) or methylphenidate (59). Notably, Hammond et al. (70) recently published a study examining the impact of amantadine on cognitive functioning in a sub-group of individuals (*n* = 119; participants were eligible if their performance on two or more neuropsychological measures fell below one standard deviation from normative means of the overall sample) from the 2015 study (63). This study found that cognitive function was not improved by amantadine (70). Psychological health, overall behavior, and social functioning improved for those administered methylphenidate (59) and carbamazepine (64). This raises the possibility that the therapeutic benefit of these drugs may not be specific to anger but rather reflect an overall lowering of psychopathology. In contrast, sertraline administration produced some limited and transient positive effects on depression, and was not associated with changes in suicidality (58). The authors concluded from this that the gains noted in aggressive behaviors and irritability could not be explained as the secondary effects of successfully treating a mood disorder (58). Collectively, these studies raise an important issue about the mechanism of action for these drugs in TBI populations, and the importance of considering comorbid factors, such as mood disorders, in prescribing practices. Finally, the impact of amantadine on global mental health was only identified by clinicians, not the participant or informant (61, 63), suggesting that clinicians were able to perceive more subtle changes in behavior and mood, which may have become apparent to participants and informants over longer follow-up periods.

### Risk of Bias

The RCTs were assessed as having low to moderate risk of bias. Most commonly, studies did not clearly report exactly how the random sequence was generated, how allocation concealment was maintained and the procedures for blinding of participants, personnel, and outcome assessors. The case series were judged to have moderate to high risk of bias and are inherently limited by lack of a control group. Across most studies, there was some limited detail provided regarding co-interventions (e.g., only providing drug class and a statement that dosage was stable during intervention), with three studies failing to provide any information regarding co-interventions (58–60). Further details about co-interventions should be provided in future studies to allow readers to determine possible synergistic or, conversely, antagonistic effects of any co-intervention. Finally, one study was supported by an “unrestricted educational grant from Pfizer, Inc.” (58).

### Summary and Implications of Review

Overall, the evidence in favor of amantadine suggests that a trial of amantadine in patients with aggression or irritability after TBI (and post PTA) may be of benefit, and should be considered in the outpatient setting. The RCT evidence for methylphenidate and propranolol was deemed insufficient to draw conclusions, and as such no recommendations are made for these medications. Likewise, the evidence from the case series examining use of carbamazepine, valproic acid, sertraline, and quetiapine is considerably limited by the study design and risk of bias, with further evidence required to formulate strong conclusions for these medications.

This review highlighted how an individual's response to medication may vary widely from the overall analysis. This is not surprising given the myriad factors that can lead to post-TBI aggression, and which may, along with other factors, impact medication metabolism and efficacy. It is possible that some of the factors influencing aggression are yet to be identified (60), and it suggested that further work should be done to identify such factors. This finding also raises the question as to whether the end point of “statistically significant” group level change is the best way to evaluate these trials. It may instead be more helpful to review the proportion of participants with “clinically meaningful” change on the primary outcome measure in the treatment and control groups, as was done by Hammond et al. (61–63).

With respect to trial design, one suggestion would be a design wherein RCTs are still used; however, when a patient fails to respond to a particular drug, they are moved (following a wash out period) to a new intervention arm with a different pharmacological intervention. The characteristics of the participants in each treatment responsive group could then be analyzed in an attempt to identify factors that suggest a person will respond to a particular drug (i.e., TBI-related factors such as area and extent of damage, presence of co-interventions, history of significant substance abuse). It is acknowledged, however, that such multi-step trials are difficult to obtain funding for and challenging to implement. Translated into clinical practice, a framework could be provided with factors or combinations of factors that should be considered when prescribing medications for post-TBI aggression, and how these might increase or decrease the likely efficacy of specific medications. This idea is consistent with growing support within the literature of a need for an individualized approach to medication in TBI patients (34, 38, 44, 45, 69). The concept of considering a range of pertinent factors in drug choice is hardly novel. However, a comprehensive evidence base to guide such decision making within the TBI population is lacking.

### Limitations

This review was limited by lack of an assessment for publication bias. However, the search strategy included a comprehensive search for unpublished studies through clinical trial registries, food and drug regulators, and correspondence with key authors. It is notable that a number of studies were excluded at full text because they failed to clearly differentiate whether patients were in the PTA period. This is important as there may be significant differences in the factors that lead to the development and maintenance of aggression in and out of PTA. For our understanding of aggression post TBI and its management to progress, study samples should be restricted to patients who are clearly in or out of PTA. Notwithstanding the noted limitations, this review represents an important systematic analysis of the evidence for pharmacotherapy for aggression post TBI that has included both efficacy and harms and used a comprehensive search strategy and analysis of methodological quality.

## Conclusions

Aggression is a potentially debilitating condition that can occur following TBI and reduce quality of life. This review concludes that a recommendation for use of amantadine to treat irritability and aggression in adults following TBI is appropriate. However, further research is needed to strengthen the evidence base, with larger sample sizes and consistent methodology across studies to allow for meta-analysis of findings. A pattern of inter-individual differences in treatment response was prominent in many studies, highlighting the possible use of “clinically meaningful change” as an alternate outcome measure, and use of trials with multiple intervention arms with participants being swapped between arms following treatment failures. Understanding the factors or constellation of factors that impact upon treatment success is a key issue to be examined in future studies, with long follow-up time points and data collection from multiple sources also recommended.

## Author Contributions

The searches, study selection, data extraction, and risk of bias were conducted by AH, FC, AJ, LP, and RB, with supervision provided by JP. AH completed the data synthesis with assistance and supervision provided by AJ and JP, respectively. AH led the drafting of the manuscript. All authors contributed to editing and reviewing of the manuscript and conception and design of this review.

### Conflict of Interest

One of the chief investigators MH has given talks on this topic for which travel and accommodation has been paid by the organizers. In addition, he has accepted fees for consulting and research from the pharmaceutical companies—Bionomics, Eli Lilly, Janssen-Cilag, Lundbeck, Novartis, Pfizer, Praxis, Servier, and Lundbeck. The remaining authors declare that the research was conducted in the absence of any commercial or financial relationships that could be construed as a potential conflict of interest.
