# Supplementary material for: The Efficacy and Harms of Pharmacological Interventions for Aggression After Traumatic Brain Injury—Systematic Review
Source: Front Neurol. 2019 Nov 29;10:1169. doi: 10.3389/fneur.2019.01169 (PMC6895752; doi:10.3389/fneur.2019.01169)
Supplement: Supplementary file 1 [file Data_Sheet_1.docx]

**Appendix 1: Search strategy**

**MEDLINE search strategy**

1. Exp Brain Hemorrhage, Traumatic/ OR Brain Injuries/ OR Brain Injury, Chronic/ OR Cerebral Hemorrhage, Traumatic/ OR Cerebrovascular Trauma/ OR Craniocerebral Trauma/ OR Diffuse Axonal Injury/ OR Exp Head Injuries, Closed/ OR Head Injuries, Penetrating/ OR Exp Intracranial Hemorrhage, Traumatic/ OR Exp Pneumocephalus/ OR (((Brain OR Cerebr$ OR Crani$ OR Crushing Skull OR Diffuse Axonal OR Head OR Hemisphere?) adj1 (Injur$ OR Trauma$)) OR ((Cerebr$ OR Crani$ OR Head) adj (Lesion? OR Wound?)) OR ((Posttraumatic OR Traumatic) adj Encephalopath$) OR (Traumatic adj (Brain OR Cerebr$)) OR Concuss$ OR DAI OR DAIs OR Pneumocephalus OR TBI OR TBIs).ti,ab.
2. "Anti-Anxiety Agents"/ OR "Anticonvulsants"/ OR "Antidepressive Agents"/ OR "Antipsychotic Agents"/ OR "Benzodiazepines"/ OR "Adrenergic Beta-Antagonists"/ OR Alprazolam/ OR Amitriptyline/ OR Amoxapine/ OR Aripiprazole/ OR Atenolol/ OR Atomoxetine Hydrochloride/ OR Benztropine/ OR Bromazepam/ OR Bupropion/ OR Buspirone/ OR Carbamazepine/ OR Chlordiazepoxide/ OR Chlorpromazine/ OR Citalopram/ OR Clomipramine/ OR Clonazepam/ OR Clopenthixol/ OR Clorazepate Dipotassium/ OR Clozapine/ OR Desipramine/ OR Desvenlafaxine Succinate/ OR Dextroamphetamine/ OR Diazepam/ OR Valproic Acid/ OR Domperidone/ OR Dothiepin/ OR Doxepin/ OR Droperidol/ OR Duloxetine Hydrochloride/ OR Estazolam/ OR Eszopiclone/ OR Flunitrazepam/ OR Fluoxetine/ OR Flupenthixol/ OR Fluphenazine/ OR Fluvoxamine/ OR Guanfacine/ OR Haloperidol/ OR Imipramine/ OR Isocarboxazid/ OR Methotrimeprazine/ OR Lithium/ OR Lithium Carbonate/ OR Lorazepam/ OR Loxapine/ OR Lurasidone Hydrochloride/ OR Methylphenidate/ OR Mianserin/ OR Midazolam/ OR Moclobemide/ OR Molindone/ OR Nitrazepam/ OR Nortriptyline/ OR Oxazepam/ OR Paliperidone Palmitate/ OR Paroxetine/ OR Phenelzine/ OR Phenobarbital/ OR Pindolol/ OR Prazepam/ OR Pregabalin/ OR Prochlorperazine/ OR Promazine/ OR Promethazine/ OR Propranolol/ OR Protriptyline/ OR Quetiapine Fumarate/ OR Remoxipride/ OR Risperidone/ OR Selegiline/ OR Sertraline/ OR Sulpiride/ OR Temazepam/ OR Thioridazine/ OR Thiothixene/ OR Tranylcypromine/ OR Trazodone/ OR Triazolam/ OR Trifluoperazine/ OR Trimipramine/ OR Venlafaxine Hydrochloride/ OR Vigabatrin/ OR ((Adrenergic adj Beta adj2 (Antagonist OR Block$)) OR Anti Anxiety OR Anti Convuls$ OR Anti Depress$ OR Anti Epilep$ OR Anti Psychotic? OR Antianxiety OR Anticonvuls$ OR Antidepress$ OR Antiepilep$ OR Antipsychotic$ OR Anxiolytic$ OR Benzodiazepine$ OR (Beta adj Block$) OR (Beta adj1 Adrenergic adj2 Block$) OR Thymoanaleptic$ OR Thymoleptic$ OR Agomelatine OR "S 20098" OR S20098 OR Thymanax OR Valdoxan OR "AGO 178" OR AGO178 OR Alprazolam OR Alprazolan OR "Apo Alpraz" OR ApoAlpraz OR Cassadan OR "D 65MT" OR D65MT OR Xanax OR Tafil OR Trankimazin OR "Novo Alprazol" OR NovoAlprazol OR "Nu Alpraz" OR NuAlpraz OR Ralozam OR "U-31,889" OR "U31,889" OR Alprox OR Esparon OR Kalma OR Amisulpride OR Sultopride OR Barnetil OR "DAN 2163" OR Solian OR "LIN 1418" OR Amitriptyline OR Amineurin OR Amitrip OR Amitriptylin OR Amitrol OR Tryptine OR ApoAmitriptyline OR Damilen OR Domical OR Laroxyl OR Endep OR Lentizol OR Novoprotect OR Saroten OR Sarotex OR Syneudon OR Triptafen OR Tryptizol OR Tryptanol OR Elavil OR Anapsique OR Amoxapine OR Desmethylloxapine OR "CL 67,772" OR "CL67,772" OR Demolox OR Asendin OR Defanyl OR Asendis OR Aripiprazole OR Aripiprazol OR "OPC 14597" OR Abilify OR Asenapine OR Saphris OR " OR G 5222" OR Atenolol OR Tenormine OR Tenormin OR "ICI 66082" OR ICI66082 OR Atomoxetine OR Tomoxetine OR Strattera OR "LY 139603" OR Benztropine OR Benzatropine OR Bensylate OR PMSBenztropine OR Cogentin OR Cogentinol OR Methylbenztropine OR ApoBenztropine OR Brexpiprazole OR Bromazepam OR BromaLich OR "Bromaz 1A Pharma" OR Bromazanil OR "Bromazep von CT" OR Durazanil OR Lexotan OR Lexotanil OR Lexatin OR Lexomil OR "Ro 5-3350" OR "Ro 53350" OR Anxyrex OR Bupropion OR Amfebutamone OR Zyntabac OR Quomen OR Wellbutrin OR Zyban OR Buspirone OR "MJ 9022 1" OR MJ90221 OR Neurosine OR Busp OR Anxut OR Buspar OR Bespar OR Carbamazepine OR Tegretol OR Carbazepin OR Epitol OR Finlepsin OR Neurotol OR Amizepine OR Cariprazine OR "RGH 188" OR Chlordiazepoxide OR Methaminodiazepoxide OR Librium OR Chlozepid OR Elenium OR Chlorpromazine OR Thorazine OR Aminazine OR Largactil OR Chlordelazine OR Contomin OR Fenactil OR Propaphenin OR Chlorazine OR Citalopram OR Cytalopram OR "Lu 10 171" OR Lu10171 OR Escitalopram OR Lexapro OR Clobazam OR "HR 376" OR Onfi OR "LM 2717" OR Frisium OR Urbanyl OR Clomipramine OR Chlomipramine OR Chlorimipramine OR Hydiphen OR Anafranil OR Clonazepam OR "Ro 5 4023" OR "Ro 54023" OR Antelepsin OR Rivotril OR Clopenthixol OR Zuclopenthixol OR Cisordinol OR Clorazepate OR Chlorazepate OR Tranxene OR Tranxilium OR "4306 CB" OR Clozapine OR Clozaril OR Leponex OR Desipramine OR Desmethylimipramine OR Demethylimipramine OR Norpramin OR Pertofrane OR Pertrofran OR Pertofran OR Petylyl OR Desvenlafaxine OR "O Desmethylvenlafaxine" OR "WY 45,233" OR "WY 45,233" OR "WY45,233" OR "WY 45233" OR WY45233 OR Pristiq OR Dextroamphetamine OR Dexamphetamine OR Dexamfetamine OR "Dextro Amphetamine" OR "D Amphetamine" OR Dexedrine OR DextroStat OR Oxydess OR Diazepam OR Diazemuls OR Faustan OR Valium OR Seduxen OR Sibazon OR Stesolid OR Apaurin OR Relanium OR "Valproic Acid" OR Divalproex OR "Propylisopropylacetic Acid" OR "2 Propylpentanoic Acid" OR Convulsofin OR Depakene OR Depakine OR Depakote OR Vupral OR Valproate OR Ergenyl OR "Dipropyl Acetate" OR Domperidone OR Domperidon OR Domidon OR Gastrocure OR Motilium OR Nauzelin OR Peridys OR "R 33,812" OR "R33,812" OR "R 33812" OR R33812 OR Dothiepin OR Dosulepin OR Prothiaden OR Doxepin OR Deptran OR Desidox OR Doneurin OR Doxepia OR Espadox OR Mareen OR Prudoxin OR Quitaxon OR Sinequan OR Sinquan OR Zonalon OR Xepin OR Aponal OR ApoDoxepin OR Droperidol OR Inapsine OR Dehidrobenzperidol OR Dehydrobenzperidol OR Droleptan OR Duloxetine OR "LY 248686" OR LY248686 OR "LY 227942" OR LY227942 OR Cymbalta OR Estazolam OR Tasedan OR ProSom OR "D 40TA" OR D40TA OR Nuctalon OR Eszopiclone OR Lunesta OR Estorra OR Flunitrazepam OR Fluridrazepam OR Flunibeta OR Flunimerck OR Fluninoc OR Rohypnol OR Rohipnol OR Narcozep OR "Flunizep von CT" OR "RO 5 4200" OR RO54200 OR Fluoxetine OR Fluoxetin OR "Lilly 110140" OR Lilly110140 OR Sarafem OR Prozac OR Flupenthixol OR Flupentixol OR Emergil OR Fluanxol OR Fluphenazine OR Flufenazin OR Lyogen OR Prolixin OR Fluvoxamine OR Fluvoxadura OR Fluvoxamin OR Fluvoxamina OR Luvox OR Fevarin OR Floxyfral OR Dumirox OR Faverin OR Desiflu OR "DU 23000" OR DU23000 OR Guanfacine OR Tenex OR Lon798 OR "BS 100 141" OR BS100141 OR Estulic OR Haloperidol OR Haldol OR Iloperidone OR Zomaril OR Fanapt OR "HP 873" OR Imipramine OR Imizin OR Norchlorimipramine OR Imidobenzyle OR Tofranil OR Melipramine OR Pryleugan OR Janimine OR Isocarboxazid OR Lamotrigine OR Crisomet OR Lamictal OR Lamiktal OR "BW 430C" OR Labileno OR Methotrimeprazine OR Levomepromazine OR Levopromazine OR Levomeprazin OR Tisercin OR Tizercine OR Tizertsin OR Lithium OR Dilithium OR Lithane OR Lithobid OR Lithonate OR "CP-15,467 61" OR "CP15,46761" OR Micalith OR "NSC 16895" OR NSC16895 OR Priadel OR "Quilinorm Retard" OR Quilinormretard OR Eskalith OR Lithotabs OR Lorazepam OR Ativan OR Temesta OR "Orfidal Wyeth" OR Donix OR Duralozam OR Durazolam OR Idalprem OR Laubeel OR "Lorazep von CT" OR "Novo Lorazem" OR NovoLorazem OR "Nu Loraz" OR NuLoraz OR Sedicepan OR Sinestron OR Somagerol OR Tolid OR "WY 4036" OR WY4036 OR ApoLorazepam OR Loxapine OR Cloxazepine OR Oxilapine OR Loxitane OR Loxipine OR Loxapinsuccinate OR "CL 71,563" OR "CL71,563" OR Lurasidone OR "SM 13496" OR SM13496 OR "SM-13,496" OR "SM13,496" OR Latuda OR Methylphenidate OR Metadate OR Equasym OR Methylin OR Concerta OR Phenidylate OR Ritalin OR Ritaline OR Tsentedrin OR Centedrin OR Daytrana OR Mianserin OR Tolvon OR Lerivon OR Org GB 94 OR Midazolam OR Dormicum OR Versed OR "Ro 21 3981" OR "Ro 213981" OR Milnacipran OR Midalcipran OR Levomilnacipran OR Savella OR "F 2207" OR Ixel OR Mirtazapine OR "6 Azamianserin" OR Esmirtazapine OR Remeron OR Remergil OR Zispin OR Norset OR Rexer OR "Org 50081" OR " OR G 3770" OR Moclobemide OR Moclobamide OR Arima OR Aurorix OR Manerix OR Moclamine OR Aurorex OR Deprenorm OR Feraken OR Moclobemid OR Moclobeta OR Moclodura OR Moclonorm OR Rimoc OR "Ro 11 1163" OR Modafinil OR Benzhydrylsulfinylacetamide OR "CRL 40476" OR Vigil OR Provigil OR Sparlon OR Alertec OR Modiodal OR Molindone OR Moban OR Nefazodone OR Rulivan OR Serzone OR Dutonin OR Nefadar OR Menfazona OR Nitrazepam OR Nitrodiazepam OR "Dormo Puren" OR Eatan OR Imadorm OR Imeson OR Mogadon OR Nitrazadon OR Nitrazep OR Novanox OR Radedorm OR Remnos OR Serenade OR Somnite OR Alodorm OR Dormalon OR Nortriptyline OR Desmethylamitriptylin OR Desitriptyline OR Aventyl OR Paxtibi OR Allegron OR Norfenazin OR Pamelor OR Nortrilen OR Olanzapine OR Zolafren OR "LY 170052" OR Zyprexa OR "LY 170053" OR Oxazepam OR Serax OR Tazepam OR Adumbran OR Oxcarbazepine OR Timox OR Trileptal OR "GP 47680" OR Paliperidone OR "9 OH Risperidone" OR "9 Hydroxy Risperidone" OR "9 Hydroxyrisperidone" OR Invega OR "R 76477" OR R76477 OR Paroxetine OR "BRL 29060" OR BRL29060 OR "FG 7051" OR FG7051 OR Seroxat OR Paxil OR Aropax OR Periciazine OR Propericiazine OR Pericyazine OR Neuleptil OR Neuleptyl OR Aolept OR Phenelzine OR "Beta Phenylethylhydrazine" OR "2 Phenethylhydrazine" OR Fenelzin OR Phenethylhydrazine OR Nardelzine OR Nardil OR Phenobarbital OR Phenobarbitone OR "Phenylethylbarbituric Acid" OR Phenemal OR Phenylbarbital OR Hysteps OR Luminal OR Gardenal OR Pindolol OR Prindolol OR Visken OR "LB 46" OR LB46 OR Prazepam OR Lysanxia OR Reapam OR Centrax OR Demetrin OR Pregabalin OR "3 Isobutyl GABA" OR Lyrica OR "CI 1008" OR CI1008 OR Prochlorperazine OR Compazine OR Promazine OR Sparine OR Sinophenin OR Protactyl OR Promethazine OR Prometazin OR Proazamine OR Rumergan OR Diprazin OR Phenergan OR Phenargan OR Phensedyl OR Pipolfen OR Pipolphen OR Promet OR Prothazin OR Pyrethia OR Remsed OR Atosil OR Diphergan OR Propranolol OR Propanolol OR Inderal OR Avlocardyl OR "AY 20694" OR AY20694 OR Rexigen OR Dexpropranolol OR Dociton OR Obsidan OR Obzidan OR Anaprilin OR Anapriline OR Betadren OR Protriptyline OR Vivactil OR Quetiapine OR "ICI 204,636" OR "ICI 204636" OR ICI204636 OR Seroquel OR Reboxetine OR Vestra OR Remoxipride OR "FLA 731" OR FLA731 OR Risperidone OR Risperdal OR Risperidal OR "R 64,766" OR "R64,766" OR "R 64766" OR R64766 OR Selegiline OR Selegyline OR "L Deprenyl" OR "E 250" OR E250 OR Eldepryl OR Emsam OR Zelapar OR Deprenil OR Deprenalin OR Yumex OR Jumex OR Humex OR Deprenyl OR Sertindole OR Serlect OR "Lu 23 174" OR Serdolect OR Sertraline OR Zoloft OR Altruline OR Lustral OR Aremis OR Besitran OR Sealdin OR Gladem OR Sulpiride OR Sulperide OR Arminol OR Deponerton OR Meresa OR Desisulpid OR Digton OR Dogmatil OR Dolmatil OR Eglonyl OR Ekilid OR Guastil OR Lebopride OR Neogama OR Pontiride OR Psicocen OR Sulp OR Sulpitil OR Sulpivert OR Sulpor OR Synedil OR Tepavil OR Aiglonyl OR Temazepam OR Hydroxydiazepam OR Methyloxazepam OR Signopam OR Tenox OR "WY 3917" OR WY3917 OR Dasuen OR Euhypnos OR Levanxol OR "Norkotral Tema" OR Normison OR Nocturne OR Temtabs OR Normitab OR Nortem OR Planum OR "Pronervon T" OR Remestan OR Restoril OR "Ro 5 5345" OR Ro55345 OR "SaH 47 603" OR "SaH 47603" OR Temaze OR "Temazep von CT" OR Thioridazine OR ApoThioridazine OR Meleril OR Melleril OR Melleryl OR Mellaril OR Melleretten OR Melzine OR Thiozine OR Sonapax OR Thioridazineneurazpharm OR Aldazine OR Rideril OR Thiothixene OR Tiotixene OR Navane OR Topiramate OR USL255 OR "McN 4853" OR Topamax OR Epitomax OR Tranylcypromine OR "Trans 2 Phenylcyclopropylamine" OR Jatrosom OR Transamine OR Parnate OR Trazodone OR Tradozone OR "AF 1161" OR AF1161 OR Deprax OR Desyrel OR Molipaxin OR Trittico OR Thombran OR "Trazodon Hexal" OR "Trazodon Neuraxpharm" OR Trazon OR Triazolam OR "U 33,030" OR "U33,030" OR Halcion OR Trilam OR "Apo Triazo" OR Trifluoperazine OR Trifluoroperazine OR Trifluperazine OR Eskazine OR Flupazine OR Terfluzine OR Triftazin OR Stelazine OR Trimipramine OR Trimeprimine OR Herphonal OR Trimineurin OR NovoTripramine OR Rhotrimine OR Stangyl OR Surmontil OR Trimidura OR Trimineurin OR Trimipramin OR "Apo Trimip" OR ApoTrimip OR Eldoral OR Venlafaxine OR "Wy 45030" OR Wy45030 OR "Wy 45,030" OR "Wy45,030" OR Effexor OR Trevilor OR Vandral OR Efexor OR Dobupal OR Vigabatrin OR "Gamma Vinyl GABA" OR "Gamma Vinyl Gamma Aminobutyric Acid" OR Sabril OR Sabrilex OR Vortioxetine OR Brintellix OR "Lu AA21004" OR LuAA21004 OR Zaleplon OR "SKP 1041" OR Sonata OR Zelepion OR Starnoc OR "CL 284,846" OR "CL284,846" OR "CL 284846" OR "L 846" OR Ziprasidone OR Ziprazidone OR "CP 88,059" OR "CP 88059" OR Zolpidem OR Amsic OR Bikalm OR Dalparan OR "SL 80.0750" OR "SL 800750 23 N" OR Stilnoct OR Stilnox OR Zodormdura OR Zoldem OR Zolirin OR "Zolpi Lich" OR Zolpinox OR Zolpimist OR Ambien OR Zopiclone OR Zop OR Zopicalma OR Zopiclodura OR Zopiclon OR Zopitan OR Zorclone OR Imovane OR Ximovan OR Zimovane OR Limovan OR Optidorm OR Rhovane OR "RP 27 267" OR Siaten OR Somnosan OR Zileze OR Zimoclone OR "Zopi Puren" OR Zopicalm OR Zotepine OR Zoleptil OR Nipolept OR Zuclopenthixol OR Zuclopentixol OR Clopixol OR Zuclopenthixole OR Acuphase).ti,ab.
3. (1 AND 2) NOT (Animals NOT (Humans NOT Animals)).sh.

Limit 3 to English
