# Supplementary material for: The Efficacy and Harms of Pharmacological Interventions for Aggression After Traumatic Brain Injury—Systematic Review
Source: Front Neurol. 2019 Nov 29;10:1169. doi: 10.3389/fneur.2019.01169 (PMC6895752; doi:10.3389/fneur.2019.01169)
Supplement: Supplementary file 2 [file Table_1.pdf]

Supplementary Table 1

Study characteristics and findings from the ten included studies

Study Details	Sample Demographics	TBI	Pharmacological Intervention & Comparator	Co-Intervention	Outcome; Follow-Up Time Points & Analyses	Findings	Study Drop Outs
**Hammond, 2017**[1]<br><br>**Parallel group, randomized, double-blind, placebo-controlled trial**<br><br>**USA; Multi-site, Rehabilitation Centres**<br><br>**Cochrane RoB**<br>**LR: 6**<br>**HR: 0**<br>**UR: 1**<br>**N/A: 0**	N: 118<br>Treatment: 61<br>Control: 57<br><br>**Sample**<br>- Closed TBI =>6mths and mod-sev aggression (=>6 on observer NPI-A)<br><br>Gender<br>Treatment<br>Males: 83.6%<br>Control<br>Males: 75.4%<br><br>Age<br>Treatment<br>Md 37.6yrs<br>Control<br>Md 35.5yrs	**Severity**<br>LoC<br>Treatment<br><24hr: 35.6%<br>1-6 days: 27.1%<br>7-29 days: 25.5%<br>=>30 days: 11.9%<br><br>Control<br><24hr: 49.1%<br>1-6 days: 15.8%<br>7-29 days: 22.8%<br>=>30 days: 12.3%<br><br>PTA<br>Treatment<br><24hr: 10.3%<br>1-6 days:12.1%<br>7-29 days: 37.8<br>>30 days: 39.7%<br><br>Control<br><24hr: 10.9%<br>1-6 days: 27.3%<br>7-29 days: 30.9%<br>=>30 days: 30.9%<br><br>GCS	**Amantadine (Anti-parkinsonian)**<br>Dose: 100mg<br>Freq: 2/day<br>Dur: 60 days<br><br>**Placebo**<br>Freq: 2/day<br>Dur: 60 days	Other:<br>- concomitant use of neuroleptics or MAOI were excluded<br>- all psychoactive meds were stable >1mth before enrollment with no plan to start/change meds during trial.	**Primary**[2]<br>**Primary - Changes in Aggression/Anger**<br>**NPI-A Most Problematic Item (observer and participant rated):**<br>Baseline, 28 days, 60 days<br>Wilcoxon rank sum test and chi-square/ fisher exact test for meaningful change analysis (>2 pt change)<br><br>**NPI-A Distress Scores**[3] **(observer and participant rated):**<br>Baseline, 28 days, 60 days<br>Wilcoxon rank sum test	[4]**Primary - Changes in Aggression/Anger**<br><br>**NPI-A Most Problematic Item:**<br>***Observer Rated***<br>- There was no statistically significant difference between the groups<br>- Day 28: treatment group mean change – 3.33; placebo group mean change – 2.70, +4.6% mean difference in % improved by >2 points (treatment 58.3%; placebo 53.7%) (all ns)<br>- Day 60: treatment group mean change – 3.91; placebo group mean change – 3.04, +14.6% mean difference in % improved by >2 points (treatment 70.2%; placebo 55.6%) (all ns)<br>***Participant Rated***<br>- There was no statistically significant difference between the groups for Day 28.<br>- Day 28: treatment group mean change – 4.15; placebo group mean change – 3.38, -1.8% mean difference in % improved by >2 points (treatment 40.0%; placebo 41.8%) (all ns)<br>- There was a statistically significant difference between the groups for Day 60.<br>- Day 60: treatment group mean change – 5.27; placebo group mean change – 2.89 (p = 0.0118 adjusted), however the mean difference of 9.2% in the % improved by > 2 points was ns (treatment 47.4%; placebo 38.2%)<br><br>**NPI-A Distress Scores:**<br>***Observer Rated***<br>- There was no statistically significant difference between the groups<br>- Day 28: treatment group mean change – 1.09; placebo group	**N: 6**<br>Due to AEs: NR<br><br>Loss to follow-up: NR

---

[1] Used a subset of the sample recruited for the Hammond 2015 study.

[2] Primary outcomes were also reported for the entire sample in supplementary materials (i.e. NPI-A, STAXI-2). Only one analysis remained significant after adjustment for multiple comparisons. NPI-A Most Problematic Item (observer rated) Day 60 (treatment group mean change – 3.01; placebo group mean change – 1.61, p = 0.0491).

[3] Only those with Distress score > 2 were included in this analysis.

[4] All findings reported here are taken from the intention to treat analyses as opposed to the per protocol analysis (excluded those with <80% pill count or failure to undergo NPI-I assessment).

	Treatment<br>3-8: 18.5%<br>9-12: 1.9%<br>13-15: 24.1%<br>Control<br>3-8: 30.8%<br>9-12: 0%<br>13-15: 28.8%		mean change – 1.15 (ns)<br>- Day 60: treatment group mean change – 1.54; placebo group mean change – 1.26 (ns)<br>***Participant Rated***<br>- There was no statistically significant difference between the groups for Day 28.<br>- Day 28: treatment group mean change – 1.97; placebo group mean change – 1.18 (ns)<br>- There was a statistically significant difference between the groups for Day 60.<br>- Day 60: treatment group mean change – 2.56; placebo group mean change – 1.44 (p = 0.0118 adjusted)
		**STAXI-2 State Anger, Trait Anger, Anger Expression (observer and participant rated):**<br>Baseline, 28 days, 60 days<br>Wilcoxon rank sum test	**STAXI-2:**<br>**State Anger**<br>***Observer Rated***<br>- There was no statistically significant difference between the groups<br>- Day 28: treatment group mean change – 2.73; placebo group mean change – 2.88 (ns)<br>- Day 60: treatment group mean change – 4.95; placebo group mean change – 0.68 (ns)<br>***Participant Rated***<br>- There was no statistically significant difference between the groups<br>- Day 28: treatment group mean change – 1.95; placebo group mean change – 2.86 (ns)<br>- Day 60: treatment group mean change – 3.24; placebo group mean change – 2.59 (ns)<br><br>**Trait Anger**<br>***Observer Rated***<br>- There was no statistically significant difference between the groups<br>- Day 28: treatment group mean change – 8.10; placebo group mean change – 9.62 (ns)<br>- Day 60: treatment group mean change – 12.91; placebo group mean change – 10.53 (ns)<br>***Participant Rated***<br>- There was no statistically significant difference between the groups<br>- Day 28: treatment group mean change – 11.51; placebo group mean change – 9.08 (ns)<br>- Day 60: treatment group mean change – 14.16; placebo group mean change – 11.68 (ns)<br><br>**Anger Expression**<br>***Observer Rated***<br>- There was no statistically significant difference between the

					**Primary – Harms**<br>Weekly<br>Fisher Exact Test<br><br>**Other Outcomes**<br>N/A<br>**Review Outcomes Not Reported**<br>Primary: N/A<br>Secondary: psychological health, cognition, QoL, participation	groups<br>- Day 28: treatment group mean change – 8.07; placebo group mean change – 6.73 (ns)<br>- Day 60: treatment group mean change – 10.88; placebo group mean change – 10.08 (ns)<br>***Participant Rated***<br>- There was no statistically significant difference between the groups for Day 28 or Day 60 (after adjustment for multiple comparisons)<br>- Day 28: treatment group mean change – 9.76; placebo group mean change – 5.78 (ns)<br>- Day 60: treatment group mean change – 13.62; placebo group mean change – 6.92 (ns)<br><br>**Primary – Harms**<br>- No significant group differences in adverse events.	
**Hammond, 2015**<br><br>**Parallel group, randomized, double-blind, placebo-controlled trial**<br><br>**USA; Multi-site, Rehabilitation Centres**<br><br>**Cochrane RoB**<br>**Cochrane RoB**<br>**LR: 6**	N: 168<br>Treatment: 82<br>Control: 86<br><br>**Sample**<br>- Closed TBI =>6mths and irritability (=>6 on observer NPI-I Most Problematic)<br><br>Gender<br>Control<br>Males: 74.4%<br>Treatment<br>Males: 80.5%<br><br>Age<br>Treatment M 40.18yrs (SD	**Severity**<br>LoC<br>Treatment<br><24hr: 35.9%<br>1-6days: 24.4%<br>7-29days: 28.2%<br>=>30days: 11.5%<br><br>Control<br><24hr: 43%<br>1-6days: 16.3%<br>7-29days: 27.9%<br>=>30days: 12.8%<br><br>PTA<br>Treatment<br><24hr: 11.5%<br>1-6 days:11.5%<br>7-29 days:34.7	**Amantadine (Anti-parkinsonian)**<br>Dose: 100mg<br>Freq: 2/day<br>Dur: 60 days<br><br>**Placebo**<br>Freq: 2/day<br>Dur: 60 days	Other:<br>- concomitant use of neuroleptics or MAOI were excluded<br>- all psychoactive meds were stable >1mth before enrollment with no plan to start/change meds during trial.	**Primary**<br>**Primary - Changes in Aggression/Anger**<br>**NPI-I Most Problematic Item (observer and participant rated):**<br>Baseline, 28 days, 60 days<br>Wilcoxon rank sum test and chi-square for meaningful change analysis (>2 pt change)	[5]**Primary - Changes in Aggression/Anger**<br><br>**NPI-I Most Problematic Item:**<br>***Observer Rated***<br>- There was no statistically significant difference between the groups<br>- Day 28: treatment group mean change – 3.69; placebo group mean change - 3.58, -.4% mean difference in % improved by >2 points (treatment 66.3%; placebo 66.7%) (all ns)<br>- Day 60: treatment group mean change – 4.68; placebo group mean change – 3.80, +6.4% mean difference in % improved by >2 points (treatment 74.7%; placebo 68.3%) (all ns)<br>***Participant Rated***<br>- There was no statistically significant difference between the groups (after adjusting for multiple comparison for Day 60).<br>- Day 28: treatment group mean change – 2.56; placebo group mean change – 1.87, +10.8% mean difference in % improved by >2 points (treatment 51.3%; placebo 49.5%) (all ns)<br>- Day 60: treatment group mean change – 3.47; placebo group mean change – 2.29, +11.7% mean difference in % improved by	**N: 11**<br>Due to AEs: NR<br><br>Loss to follow-up: NR

[5] All findings reported here are taken from the intention to treat analyses as opposed to the per protocol analysis (excluded those with <80% pill count or failure to undergo NPI-I assessment).

**HR: 0**<br>**UR: 1**<br>**N/A: 0**	12.67)<br>Control<br>M 38.23yrs (SD 12.36)	=>30 days: 42.3%<br><br>Control<br><24hr: 9.6%<br>1-6 days: 22.9%<br>7-29 days: 26.5%<br>=>30 days: 41%<br><br>GCS<br>Treatment<br>3-8: 22.5%<br>9-12: 4.2%<br>13-15: 23.9%<br>Control<br>3-8: 30.8%<br>9-12: 1.3%<br>13-15: 25.6%			>2 points (treatment 60.5%; placebo 48.8%) (all ns)
				**NPI-I Most Aberrant Item (observer and participant rated):**<br>Baseline, 28 days, 60 days<br>Wilcoxon rank sum test<br>Wilcoxon rank sum test and chi-square for meaningful change analysis (>2 pt change)	**NPI-I Most Aberrant Item:**<br>***Observer Rated***<br>- There was no statistically significant difference between the groups<br>- Day 28: treatment group mean change – 3.74; placebo group mean change – 3.68 (ns), -10.4% mean difference in % improved by >2 points (treatment 60.0%; placebo 70.4%) (all ns)<br>- Day 60: treatment group mean change –4.39; placebo group mean change – 3.90 (ns), -.3% mean difference in % improved by >2 points (treatment 68.0%; placebo 68.3%) (all ns)<br>***Participant Rated***<br>- There was no statistically significant difference between the groups<br>- Day 28: treatment group mean change – 2.98; placebo group mean change – 1.87, -10.4% mean difference in % improved by >2 points (treatment 60.0%; placebo 70.4%) (all ns)<br>- Day 60: treatment group mean change –3.70; placebo group mean change – 2.77 (ns), +6.9% mean difference in % improved by >2 points (treatment 60.5%; placebo 53.6%) (all ns
				**NPI-I Distress Scores (observer and participant rated):**<br>Baseline, 28 days, 60 days<br>Wilcoxon rank sum test	**NPI-I Distress Scores:**<br>***Observer Rated***<br>- There was no statistically significant difference between the groups<br>- Day 28: treatment group mean change – 1.38; placebo group mean change – 1.03 (ns)<br>- Day 60: treatment group mean change – 1.62; placebo group mean change – 1.33 (ns)<br>***Participant Rated***<br>- There was no statistically significant difference between the groups (after adjusting for multiple comparison for Day 60).<br>- Day 28: treatment group mean change – 1.52; placebo group mean change – 1.17 (ns)<br>- Day 60: treatment group mean change – 1.87; placebo group mean change – 1.35 (ns)
				**Primary – Harms**<br>Weekly<br>Fisher Exact Test	**Primary – Harms**<br>- No significant group differences in adverse events.

					**Secondary – Psychological Health**<br>**Global Impression of Change (observer and participant rated):**<br>Baseline, 28 days, 60 days<br>Analysis unclear<br><br>**Clinical Global Impressions – Global Improvement subscale (clinician rated):**<br>Baseline, 28 days, 60 days<br>Analysis unclear<br><br>**Other Outcomes**<br>N/A<br>**Review Outcomes Not Reported**<br>Primary: N/A<br>Secondary: cognition, QoL, participation	**Secondary – Psychological Health**<br>**Global Impression of Change:**<br>***Observer Rated***<br>- Despite large improvements in both groups, there was no statistically significant difference between the groups<br>***Participant Rated***<br>- There was no statistically significant difference between the groups<br><br>**Clinical Global Impressions:**<br>- At 28 days, there was no statistically significant difference between the groups<br>- At 60 days, there was greater global improvement for the treatment group (M 2.65, SD 1.05) then the placebo group (M 3.01 SD 1.08) (p = 0.035)	
**Hammond, 2014**<br><br>**Parallel group, randomized, double-blind, placebo-controlled trial**<br><br>**USA; Rehabilitation Centre**<br><br>**Cochrane RoB**<br>**LR: 4**<br>**HR: 0**<br>**UR: 3**<br>**N/A: 0**	N: 76<br>Treatment: 38<br>Control: 38<br><br>**Sample**<br>- Closed TBI =>6mths and irritability (score > 2 NPI-I)<br><br>Gender<br>Treatment<br>Males: 65.79%<br>Control<br>Males: 57.89%<br><br>Age<br>Treatment<br>M 34.7yrs (SD 13.2)<br>Control<br>M 42.1yrs (SD 13.7)	**Time post injury**<br>Treatment<br>M 5.3yrs (SD 6)<br>Control<br>4.7yrs (SD 4.2)<br><br>**Severity**<br>GCS<br>Treatment<br>M 9.5 (SD 4.4.)<br>Control<br>M 7.5 (SD 5.1)	**Amantadine (Anti-parkinsonian)**<br>Dose: 100mg<br>Freq: 2/day<br>Dur: 28 days<br><br>**Placebo**<br>Freq: 2/day<br>Dur: 28 days	Other:<br>- concomitant use of neuroleptics or MAOI were excluded<br>- all psychoactive meds were stable >1mth before enrollment with no plan to start/change meds during trial.	**Primary**<br>**Primary - Changes in Aggression/Anger**<br>**NPI-I (observer rated):**<br>Baseline, 28 days<br>Wilcoxon rank sum test and chi-square test for meaningful change analysis (>2 pt change)<br><br>**NPI-I Distress (observer rated):**<br>Baseline, 28 days<br>Wilcoxon rank sum test<br><br>**NPI-I Most Problematic Item (observer rated):**<br>Baseline, 28 days<br>Wilcoxon rank sum test for frequency and severity<br><br>**NPI-A (observer rated):**<br>Baseline, 28 days<br>Wilcoxon rank sum test (note: this analysis was performed on the full sample and on a subset with NPI-	**Primary - Changes in Aggression/Anger**<br><br>**NPI-I:**<br>- There was a statistically significant difference between the groups<br>Day 28: treatment group mean change – 4.3; placebo group mean change – 2.6 (p=0.0085)<br>Day 28: +37% mean difference in % improved by >2 points (treatment 81%; placebo 44%) (p=0.0016)<br><br>**NPI-I Distress:**<br>- There was no statistically significant difference between the groups<br>- Day 28: treatment group mean change – 7.6; placebo group mean change – 5.8 (ns)<br><br>**NPI-I Most Problematic Item:**<br>- There was a statistically significant difference between the groups for the mean change in severity and frequency. Mean change values NR, but significance values provided for frequency (p = 0.0156) and severity (p = 0.0055).<br><br>**NPI-A:**<br>***Full Sample***<br>- There was no statistically significant difference between the change scores for the groups. No numerical findings were reported.	**N:4**<br>Due to AEs: 1<br><br>Loss to follow-up: 3

A>2)	***Restricted Sample (NPI-A>2 at Baseline; n = 54)***<br>- There was a statistically significant difference between the groups<br>- Day 28: treatment group mean change – 4.56; placebo group mean change – 2.46 (p=0.046)
**NPI-A Distress (observer rated):**<br>Baseline, 28 days<br>Wilcoxon rank sum test	**NPI-A Distress:**<br>- There was no statistically significant difference between the change scores for the groups. No numerical findings were reported.
**Primary – Harms**[6]<br>4 days, 14 days, 28 days<br>Chi-square/ fisher exact test for difference in proportions<br>Wilcoxon rank sum test for difference in severity of event	**Primary – Harms**<br>- No significant group differences in proportion or severity of adverse events.<br>- One participant required study drug termination secondary to a seizure.<br>- No dose reduction was required.
**Secondary – Psychological Health**<br>**BDI-II:**<br>Baseline, 28 days<br>Wilcoxon rank sum test	**Secondary – Psychological Health**<br>**BDI-II:**<br>- There was no statistically significant difference between the change scores for the groups. No numerical findings were reported.
**Global Mental Health Scale:**<br>Baseline, 28 days<br>Wilcoxon rank sum test	**Global Mental Health Scale:**<br>- There was no statistically significant difference between the change scores for the groups. No numerical findings were reported.
**Brief Symptom Inventory (BSI-Anxiety):**<br>Baseline, 28 days<br>Wilcoxon rank sum test	**Brief Symptom Inventory (BSI-Anxiety):**<br>- There was no statistically significant difference between the change scores for the groups. No numerical findings were reported.
**Other Outcomes**<br>N/A	
**Review Outcomes Not Reported**<br>Primary: N/A<br>Secondary: cognition, QoL, participation	

[6] Adverse events were defined as any unfavourable and unintended diagnosis, sign (including an abnormal lab finding), symptom, or disease that occurred during study participation, whether or not related to the intervention. Adverse events include new events not present during the pre-intervention period or events that were present during the pre-intervention period but increased in severity during study participation.

**Brossart, 2008**<br><br>**RCT; double blind crossover**[7]<br><br>**USA; Rehabilitation Unit**<br><br>**Cochrane RoB**<br>**LR: 1**<br>**HR: 0**<br>**UR: 6**<br>**N/A: 0**	N: 13<br><br>**Sample**<br>- Closed or penetrating TBI > 1yr and significant agitation<br><br>Male: 69%<br><br>Age<br>M 34yrs (SD 9.78)	**Time post injury**<br>> 1yr<br><br>**Severity**<br>NR	**Propranolol (beta blocker)**<br>Dose[8]:<br>Initial: 60mg (n=8); 80mg (n=2); dosage was increased for those who tolerated medication<br>Max: 60mg (n=2), 80mg (n=6), 120mg (n=1), 180mg (n=1)<br>Freq: 1/day<br>Dur: Unclear<br><br>**Placebo**<br>Freq: 1/day<br>Dur: Unclear	NR	**Primary - Changes in Aggression/Anger**<br>**ABS:**<br>Baseline: during two week baseline period; M 5.3 data points per patient (R 3-8)<br>Treatment phase: Weekly for M 10wks (R 6 – 14wks); M 5.1 data points per patient (R 3-8)<br>Logistic regression; prediction accuracy represented by Pearson's phi index of association[9]<br>Ordinary least squares multiple regression	**Primary - Changes in Aggression/Anger**<br><br>**ABS:**<br>- Classification accuracy of entire data set was 56.7%, indicating that any given data point had an equal chance of belonging to the baseline vs treatment condition, and representing an "unsuccessful intervention".<br>- Pearson's phi index was 0.135 (90% exact CI -0.03 < 0.135 < 0.29).<br>- This indicates that across the 10 participants, the magnitude of change from baseline to intervention phases was 0.135. This can be interpreted as 'small to negligible'[10]<br>- Individual analysis revealed three groups of response type (little or no effect vs moderate to strong effect – improvement vs moderate to strong effect – worsening)<br><br>Patient Number (Classification rate, Pearson's phi index (p value, 90% CI), R^2)<br>Group: little or no effect<br>Patient 3: 54.5%; 0.04 (p= 0.89, -0.38 < 0.03 < 0.46), 0.02<br>Patient 6: 50%; 0.0 (p= 1.0, -0.40 < 0.00 < 0.40), 0.07<br>Patient 7: 50%; 0.0 (p= 1.0, -0.40 < 0.00 < 0.40), 0.04<br>Patient 8 : 54.5%, 0.07 (p= 0.82, -0.40 < 0.07 < 0.53), 0.05<br>Patient 9 : 66.7%, 0.33 (p= 0.41, -0.33 < 0.33 < 0.81), 0.22<br>Patient 10 : 62.5%, 0.00 (p= 1.0, -0.40 < 0.00 < 0.54), 0.02<br><br>Group: moderate to strong effect – improvement<br>Patient 2: 100%, 1.0 (p< 0.001, 0.56 < 1.00 < 1.00), 0.7<br>Patient 5: 92.9%, 0.87 (p = 0.001, 0.44 < 0.88 < 0.99), 0.73<br><br>Group: moderate to strong effect – worsening<br>Patient 1: 80%, 0.52 (p= 0.97, -0.04 < 0.52 < 0.86), 0.23<br>Patient 4: 83.3%, 0.625 (p = 0.03, 0.09 < 0.63 < 0.88), 0.70	**N: 3**<br><br>Due to AEs: 0<br><br>Other: 3; excluded as less than 2 data points
					**Primary – Harms**<br>**Pulse & Blood Pressure:**<br>Each clinic visit (unclear when these occurred)	**Primary – Harms**<br>**Pulse & Blood Pressure:**<br>NR<br>**Other**<br>- Agitation became worse for two patients	

[7] Each participant acted as their own control.

[8] Dose of the study drug was adjusted to a tolerated dosage increment for supine blood pressure less than 55 diastolic or 95 systolic in patients under 50 years of age; less than 70 diastolic or 110 systolic in patients 50 years of age and over.

[9] Study examined LR prediction accuracy. LR predicts membership of each data point in either baseline or intervention phase (presented in a 2 x 2 table), based on its relative magnitude (with chance level being 50% accuracy). The 2 x 2 table agreement table, when analysed by using chi-square, yields the Pearson's phi index of association, which is a measure of effect size. Pearson's phi index can be interpreted approximately as 'prediction accuracy beyond chance'.

[10] Guidelines for interpreting phi magnitudes were taken from 73. Parker, R.I. and S. Hagan-Burke, *Median-based overlap analysis for single case data: a second study.* Behav Modif, 2007. **31**(6): p. 919-36.

					**Other Outcomes**<br>N/A<br><br>**Review Outcomes Not Reported**<br>Primary: N/A<br>Secondary: Psychological Health, Cognition, QoL, Participation		
**Mooney, 1993**<br><br>**RCT; single blind**<br><br>**USA; Rehabilitation Centre and local community referrals**<br><br>**Cochrane RoB**<br>**LR: 2**<br>**HR: 0**<br>**UR: 5**<br>**N/A: 0**	N: 38<br>Treatment: 19<br>Control: 19<br><br>**Sample**<br>- Males with serious TBI (Loc=>6hrs/ PTA=>24hrs)<br><br>Gender<br>Male: 100%<br><br>Age<br>M 29.45yrs (SD 10.02, R 18 – 50)	**Time post injury**<br>M 27.08mths (SD 21.13)<br><br>**Severity**<br>LoC<br>M 16.74 days (SD 21.03)<br><br>PTA<br>M 56.58 days (SD 50.08)	**Methylphenidate (Stimulant)**<br>Dose:<br>Wks 1 -4: NR[11]<br>Wks 5-6: 30mg<br>Freq: 1/day<br>Dur: 6 weeks<br><br>**Placebo**<br>Freq: 1/day<br>Dur: 6 weeks	NR	**Primary - Changes in Aggression/Anger**<br>**STAS-T:**<br>Baseline, 6 weeks<br>Repeated measures ANCOVA – controlling for baseline score on STAS-T<br><br>**STAS-S:**<br>Baseline, 6 weeks<br>Repeated measures ANOVA<br><br>**POMS-Anger Hostility:**<br>Baseline, 6 weeks<br>Repeated measures ANOVA<br><br>**KAS-Belligerence:**<br>Baseline, 6 weeks<br>Repeated measures ANOVA<br><br>**Combined Anger Outcome Measures:**<br>Hierarchical clustering and discriminant analysis	**Primary - Changes in Aggression/Anger**<br>**STAS-T**<br>- Significant difference between change scores (from baseline to 6mths) in the treatment and placebo groups ($p< 0.001$).<br>- Treatment group showed a reduction (M -9.47) from baseline (M 33.58; SD 11.06) to 6 weeks (M 24.11; SD 5.69), compared to an increase (M 2.37) in the placebo group from baseline (M 26.47; SD 7.83) to 6 weeks (M 28.84; SD 8.83)<br><br>**STAS-S**<br>- Significantly greater reduction (M -4.05) in the treatment group from baseline (M 22.47; SD 8.11) to 6 weeks (M 18.42; SD 7.17), compared to the placebo group (M 0.05) from baseline (M 20.42; SD 10.06) to 6 weeks (M 20.37; SD 6.70) ($p=0.06$)<br><br>**POMS-Anger Hostility**<br>- Significantly greater reduction of M -8.42 from baseline (M 15.79; SD 10.93) to 6 weeks (M 7.37; SD 6.11) in the treatment group, compared to a decrease of only M -0.21 in the placebo group from baseline (M12.42; SD 11.22) to 6 weeks (M 12.21; SD 11.06) (($p= 0.001$)<br><br>**KAS-Belligerence**<br>- Significant difference between change scores (from baseline to 6mths) in the treatment and placebo groups ($p=0.005$).<br>- Treatment group showed a reduction (M -1.6) from baseline (M 6.79; SD 2.32) to 6 weeks (M 5.19; SD 1.68), compared to an increase (M .26) in the placebo group from baseline (M 5.95; SD 1.99) to 6 weeks (M 6.21; SD 2.25)<br><br>**Combined Anger Outcome Measures:**<br>- Hierarchical clustering produced two clusters within the treatment group<br>- The two groups were the ‘non responders’ (no reduction in anger scores from baseline to 6 weeks) and the ‘response group’ (all members exhibited clear reduction in anger from baseline to 6 weeks)	**N: 0**

[11] The medication schedule was designed so that subjects gradually built up the amount of medication taken over the first four weeks of the study and then remained as the final total daily medication dose of 30mg/day during the fifth and sixth weeks of the study.

	- Discriminant analysis revealed that participants with higher baseline anger scores were more likely to respond to the drug than participants with low baseline anger scores<br>- Participants in the ‘response group’ also showed greater change from baseline to 6 weeks for OSSI-I (M -138.57; SD 60.34 vs M -35.33; SD 78.07), OSSI-P (M -155.14; SD 82.76 vs M -21.58; SD 54.80), KAS-General Psychopathology (M -15.29; SD 5.85 vs M -4.25; SD 4.63) and the Selective Reminding Test (M 12.14; SD 10.54 vs M -4.33; SD 15.07) compared to the ‘non-responders’
**Primary - Harms**<br>**The Recent Experience Checklist:**<br>Baseline, 6 weeks<br>Repeated measures MANOVA	**Primary – Harms**<br>**The Recent Experience Checklist**<br>- No significant drug by time interaction effect was found, $F(1,36) = 1.41$, $p>0.05$
**Secondary – Psychological Health**<br>**KAS-General Psychopathology:**<br>Baseline, 6 weeks<br>Repeated measures ANOVA	**Secondary – Psychological Health**<br>**KAS-General Psychopathology**<br>- Significantly greater reduction of M -8.32 from baseline (M 46.37; SD 7.88) to 6 weeks (M 38.05; SD 3.95) in the treatment group, compared to a decrease of only M -0.31 in the placebo group from baseline (M 41.69; SD 9.57) to 6 weeks (M 41.38; SD 8.76) ($p<0.001$)
**OSSI-I:**<br>Baseline, 6 weeks<br>Repeated measures ANOVA	**OSSI-I**<br>- Significantly greater reduction of M -73.37 from baseline (M 342.63; SD 85.86) to 6 weeks (M 269.26; SD 70.97) in the treatment group, compared to a decrease of only M -1.62 in the placebo group from baseline (M 306.81; SD 78.50) to 6 weeks (M 305.19; SD 80.09) ($p=0.001$)
**OSSI-P:**<br>Baseline, 6 weeks<br>Repeated measures ANOVA	**OSSI-P**<br>- Significantly greater reduction of M -70.79 from baseline (M 331.53; SD 101.88) to 6 weeks (M 260.74; SD 106.61) in the treatment group, compared to a decrease of only M -3.97 in the placebo group from baseline (M 262.91; SD 101.76) to 6 weeks (M 258.94; SD 93.28) ($p=0.003$)
**Secondary – Cognition**<br>**Letter Cancellation Test & Selective Reminding Test:**<br>Baseline, 6 weeks<br>Repeated measures MANOVA	**Letter Cancellation Test & Selective Reminding Test**<br>- No significant drug by time interaction effect was found, $F(3,34) = 0.086$, $p>0.05$.
**Other Outcomes**<br>N/A<br>**Review Outcomes Not Reported**<br>Primary: N/A<br>Secondary: QoL; participation	

**Kim, 2006**<br><br>**Open trial case series**<br><br>**USA; Outpatient clinic**<br><br>**JBI RoB[12]**<br>**Y: 5**<br>**N: 0**<br>**U: 5**<br>**N/A: 0**	N: 7<br><br>**Sample**<br>- Individuals with TBI and irritability/ aggression that started post TBI and persisted => 1mth<br><br>Male: 57.1%<br><br>Age<br>M 48.9yrs (SD 2.4)	**Time post injury**<br>M 23.1mths (SD 15.9)<br><br>**Severity**<br>Severe: 2/7<br>Coma R 5-20 days; intracranial hemorrhage<br><br>Severity of other 5 patients NR	**Quetiapine (Atypical antipsychotic)**<br>Dose:<br>Initial (week 1): R 50mg – 100mg[13]<br>Max:<br>M 110.7mg; SD 93.4mg; R 25mg–300mg<br>Freq: 1 x day<br>Dur: 6 weeks	**Antidepressants**<br>**Benzodiazepines**<br>**Anticonvulsants**<br>N: NR<br>Dose: NR – but dose must be stable for => 2mths prior to study<br>Freq: NR<br>Dur: NR<br>Other: medication must have preceded study and not impacted aggression during that time	**Primary - Changes in Aggression/Anger**<br>**OAS-M:**<br>Time points NR<br>Paired t-test<br><br>**CGI:**<br>Time points NR<br>Paired t-test<br><br>**NFI (Aggression subscale):**<br>Time points NR<br>Paired t-test; last observation carried forward analysis for missing data<br><br>**Primary – Harms**<br>**Simpson Angus Scale;**<br>**Barnes Akathisia Rating Scale;**<br>**Abnormal Involuntary Movement Scale:**<br>Time points NR<br>Descriptive<br><br>**Secondary – Cognition**<br>**RBANS:**<br>Time points NR<br>Paired t-test<br><br>**Other Outcomes**<br>N/A<br>**Review Outcomes Not Reported**<br>Primary: N/A<br>Secondary: psychological health, QoL, participation	**Primary - Changes in Aggression/Anger**<br><br>**OAS-M:**<br>- Significant mean reduction in scores of 84.5% (p = 0.002).<br><br>**CGI:**<br>- Significant improvement from baseline (M 4.14; SD 0.38) to the end of the study period (M 2.29; SD 1.11) (p = 0.002).<br><br>**NFI (Aggression subscale):**<br>- Significant improvements over the study period (p = 0.036).<br><br>**Primary – Harms**<br>**Simpson Angus Scale;**<br>**Barnes Akathisia Rating Scale;**<br>**Abnormal Involuntary Movement Scale:**<br>- Sedation was reported in 3/7 patients (42.8%). This resolved for two patients by week 3; and for one patient by week 6.<br>- One patient had mild extrapyramidal side effects and akathisia.<br><br>**Secondary – Cognition**<br>**RBANS:**<br>- Significant mean improvement over the study period of 8.02% (p = 0.027).	**N: 0**
**Azouvi, 1999**<br><br>**Prospective open trial case series**<br><br>**France; Rehabilitation Unit**	N: 10<br><br>**Sample**<br>- Patients with severe TBI (GCS<=8) and behavioral changes	**Time post injury**<br>M 58wks (SD 59.9; R 11 – 188wks)<br><br>**Severity**<br>GCS<br>M 5.3 (SD1.6;	**Carbamazepine (Anti-Convulsant)**<br>Dose:<br>Initial: 200mg; increased by 200mg every 4days<br>Max: 600-	**Neuroleptics**<br>N: 5<br>Dose: NR<br>Freq: NR<br>Dur: NR	**Primary - Changes in Aggression/Anger**<br>**NRS – R (6 target items; hyperactivity-agitation, mood lability, irritability, disinhibition, excitation, hostility):**<br>Baseline, every 2wks<br>Wilcoxon signed rank test<br>Individual Analysis	**Primary - Changes in Aggression/Anger**<br><br>**NRS – R (6 target items):**<br>- Significant improvement from baseline (M 9.0, SD 2.0) to 8 weeks (M 4.6; SD 4.2) (tied z = -2.3, p = 0.02).<br>- Item level analysis showed that improvement was significant at 8 weeks for irritability (tied z = -2.4, p = 0.01) and disinhibition (tied z = -2.04, p < 0.05). Improvement was not significant for hyperactivity-agitation, mood lability, excitation or hostility (R	**N:1**<br><br>Due to AEs:<br>**1**

[12] Y – low risk of bias; N – high risk of bias; U – unclear risk of bias; N/A – item not applicable for study.

[13] Medication was titrated every 3-4 days as tolerated in single doses to the maximum dose by the end of week 3. This was followed by a maintenance phase of 3 weeks, during which the dose could be adjusted based on clinical need and tolerability.

**JBI RoB**<br>**Y: 7**<br>**N: 0**<br>**U: 3**<br>**N/A: 0**	Gender<br>Male: 80%<br><br>Age<br>M 33.7yrs (SD 14.8, R 22 – 71)	R 4 -8)[14]	800mg[15]<br>End of trial:<br>9.47+- 2.9 mg/kg/day<br>Freq: 1/day<br>Dur: 8wks		tied z values -1.6 to 0.37, p > .01).<br>- There was inter-individual variability in treatment response; 5 patients showed a decrease from baseline to 8wks of 50% or more, 3 patients scores decreased between 25-43%, and 2 patients showed no change.<br>- On the basis of visual analysis, responders and non-responders could not be differentiated based on either time since injury or drug dosage.
				**ABS:**<br>Baseline, every 2wks<br>Wilcoxon signed rank test	**ABS:**<br>- Significant improvement from baseline (M 32.7, SD 8.2) to 8 weeks (M 24.4; SD 9.0) (tied z = -2.2, p = 0.02).
				**Primary – Harms**<br>**Blood Samples:**<br>Every 2wks<br>Descriptives	**Primary – Harms**<br>**Blood Samples:**<br>- SAE: significant allergic cutaneous reaction (n = 1) occurred on day 51 of the intervention and required withdrawal of medication.<br>- Drowsiness (n=4) occurred at beginning of treatment resulting in lowering of medication dose.<br>- No modification of blood cell count of hepatic function was found.
				**Secondary – Cognition**<br>**MMSE:**<br>Every 2wks<br>Wilcoxon signed rank test	**Secondary – Cognition**<br>**MMSE:**<br>- No significant change from baseline (M 24.2; SD 8.1) to 8 weeks (M 25.2; SD 5.1) (tied z = -.67, p > 0.1).
				**Other Outcomes – Overall Behavior**<br>**NRS –R (full scale):**<br>Baseline, every 4wks<br>Wilcoxon signed rank test	**Other Outcomes – Overall Behavior**<br>**NRS –R (full scale):**<br>- Significant improvement from baseline (M 9.0, SD 2.05) to 8 weeks (M 5.1; SD 4.2) (p = 0.01).
				**Other Outcomes -**<br>**Social Functioning**<br>**KAS:**<br>Baseline, every 4wks<br>Wilcoxon signed rank test	**Other Outcomes -**<br>**Social Functioning**<br>**KAS:**<br>- Total number of questions for all patients for which an abnormality was described as ‘frequent’ decreased from baseline (n=219) to the last assessment (n = 131) (p < 0.01).
				**Review Outcomes Not Reported**<br>Primary: N/A<br>Secondary: psychological health;	

[14] GCS data missing for two participants so unclear how severity of injury was determined.

[15] Max dosage was individually adjusted according to efficacy and occurrence of adverse events.

					QoL; participation		
**Kant, 1998**<br><br>**Open trial case series**<br><br>**USA; Outpatient clinic**<br><br>**JBI RoB**<br>**Y: 5**<br>**N: 0**<br>**U: 5**<br>**N/A: 0**	N: 13<br><br>**Sample**<br>- History of TBI and current irritability/ aggression<br><br>Male: 77%<br><br>Age<br>M 37.6yrs (SD NR; R 20 – 57)	**Time post injury**<br>M 2yrs (SD NR; R 1mth – 9yrs)<br><br>**Severity**<br>Determined by LoC<br>Mild: 38.4%<br>Moderate: 46.2%<br>Severe: 15.4%	**Sertraline (SSRI)**<br>Dose:<br>Initial: 50mg<br>Max: Titrated to 200mg or maximum tolerable dose[16]<br>Freq: 1/day<br>Dur: 8wks	NR	**Primary - Changes in Aggression/Anger**<br>**OAS-M (Aggression, Irritability, scales):**<br>Baseline, every 2 weeks<br>T-test<br>'Clinical Improvement' (defined as post treatment raw scores that differed by .5 or more of the SD of baseline scores)	**Primary - Changes in Aggression/Anger**<br><br>**OAS-M (Aggression, Irritability, scales):**<br>Aggression scale:<br>- Significant improvement from baseline to 4 week follow-up ($t(12) = 4.32$, $p < 0.01$) and 8 week follow-up ($t(9) = 3.75$, $p < 0.01$).<br>- 10/13 patients had clinically significant decrease in scores at 4 week follow-up<br>- 8/10 patients had clinically significant decrease in scores at 4 week follow-up<br>Irritability scale:<br>- Significant improvement from baseline to 4 week follow-up ($t(12) = 5.12$, $p < 0.01$) and 8 week follow-up ($t(9) = 6.0$, $p < 0.01$).<br>- 12/13 patients had clinically significant decrease in scores at 4 week follow-up<br>- 10/10 patients had clinically significant decrease in scores at 4 week follow-up	**N: 3**<br><br>Due to AEs: NR<br><br>Other: NR
					**Primary – Harms**<br>**Clinical Assessment NOS:**<br>Each f/up visit (unclear when these occurred)	**Primary – Harms**<br>**Clinical Assessment NOS:**<br>- States in Methods section that medication "adjusted or discontinued, as indicated" if side effects identified, however, not clear if this occurred.	
					**Secondary – Psychological Health**<br>**OAS-M (Suicidality scale):**<br>Baseline, every 2 weeks<br>T-test<br>'Clinical Improvement' (as defined above)[17]	**Secondary – Psychological Health**<br>**OAS-M (Suicidality scale):**<br>- No significant change from baseline to 4 week follow-up ($t(12) = 1.76$, $p = 0.10$) and 8 week follow-up ($t(9) = 1.0$, $p = 0.34$).	
					**BDI:**<br>Baseline, every 2 weeks<br>T-test	**BDI:**<br>- Significant improvement from baseline to 4 week follow-up ($t(11) = 2.34$, $p = 0.04$)<br>- No significant change from baseline to 8 week follow-up ($t(8) = 1.63$, $p = .14$).	
					**Other Outcomes**<br>N/A<br>**Review Outcomes Not Reported**<br>Primary: N/A<br>Secondary: Cognition, QoL, Participation		

[16] Titration of dosage was carried out depending on symptom relief as reported by the patient and family members.

[17] Results for this analysis are not presented as 10/13 patients scored 0 on this measure as baseline. As such, the lack of clinically significant change actually reflect the absence of suicidality at baseline, not the lack of change in scores.

**Wroblewski, 1997**<br><br>**Open trial case series**<br><br>**USA; Rehabilitation Unit**<br><br>**JBI RoB**<br>**Y: 4**<br>**N: 1**<br>**U: 5**<br>**N/A: 0**	N: 2[18]<br><br>**Sample**<br>- TBI and behavior dyscontrol refractory to medication<br><br>Patient 1: restless, impulsive, irritable, LFT, assaultive, verbal abusiveness<br>Patient 2: destructive behaviors interfering with rehabilitation, physical and verbal aggression, property destruction<br><br>Gender<br>Patient 1: Male<br>Patient 2: Female<br><br>Age<br>Patient 1: 34yrs<br>Patient 2: 29yrs	**Time post injury**[19]<br>Patient 1: approx. 5yrs<br>Patient 2: 5yrs<br>**Severity**<br>Patient 1:<br>R subdural hematoma with severe DAI<br>Coma: NR<br><br>Patient 2:<br>R frontal subdural hematoma, subarachnoid hemorrhage, bilateral frontal contusions<br>Coma: approx. 2mths<br><br>**Previous TBI**<br>Patient 1: 2<br>Patient 2: NR	**Valproic Acid (Anti-Convulsant)**<br><br>Patient 1:<br>Initial<br>Dose: 750mg<br>Freq: 1/day<br>(serum concentration = 60µg/mL<br><br>- Text describes serum concentration and dosage changes. However, not clear when these were made and what the dosage change was.<br><br>Dur: 3 months (this is the period of data collection; patients did not discontinue treatment at end of study period)<br><br>Patient 2:<br>Dose<br>Initial: 500mg<br>Max: 1500mg<br>Freq: 1/day<br>Dur: 6wks	- Not explicitly reported but does state that all previous medication trialed to treat behavior problems had been discontinued in both patients prior to beginning intervention.	**Primary - Changes in Aggression/Anger**<br><br>**Patient 1**<br>**Count of episodes of verbal abuse, yelling, threat of assault, time out**<br>During intervention period<br>Duration of observation:<br>Baseline = 3mths, with recordings at 1mth, 2mths<br>Treatment = 3mths, with recordings at 4mths, 5mths, 6mths<br>Descriptives<br><br>**Patient 2**<br>**Count of episodes of physical aggression and time outs for verbal aggression**[20]<br>During intervention period<br>Duration of observation:<br>Baseline = 2 wks, with recordings daily<br>Treatment = 6wks, with recordings daily<br>Descriptives<br><br>**Primary – Harms**<br>During intervention period<br>Descriptives	**Primary - Changes in Aggression/Anger**<br><br>**Patient 1**<br>**Count of episodes of verbal abuse, yelling, threat of assault, time out**[23]<br>- Verbal Abuse: Decline from control period (38, 25) to the treatment period (23, 5, 5)<br>- Yelling: Decline from control period (60, 34) to the treatment period (23, 10, 5)<br>- Threat of assault: Results in figure are not clear for control period. Decline during the treatment period (23, 5, 5)<br>- Time Out: Decline from control period (29, 32) to the treatment period (23, 5, 5)<br><br>**Patient 2**<br>**Count of episodes of physical aggression and time outs for verbal aggression**<br>Physical Aggression<br>- Control period ranged from 0 to 14 responses per day<br>- Treatment period was reported for each dose:<br>- 500mg/day: 0 -3 responses per day<br>- 750mg/day: 0-2 responses per day<br>- 10000mg/day: 0-6 responses per day<br>-1250mg/day: 0-3 responses per day<br>- 1500mg/day: 0 responses per day; maintained for 14 consecutive days<br>Time Outs<br>- Control period ranged from 0 to 8 time outs per day<br>- Treatment period was reported for each dose:<br>- 500mg/day: 0 -3 time outs per day<br>- 750mg/day: 0-3 time outs per day<br>- 10000mg/day: 0-3 time outs per day<br>-1250mg/day: 0-3 time outs per day<br>- 1500mg/day: 0 time outs per day; maintained for 14 consecutive days<br><br>**Harms**<br>- No observable or notable adverse effects ether systematically or cognitively in either patient.	**N: 0**<br>Due to AEs: NR<br><br>Loss to follow-up: NR

[18] The paper includes data on 5 individuals. Three individuals were excluded due to (1) not TBI, (2) no quantitative data provided and (3) possibly in PTA - this patient was described as being in the PTA period towards the beginning of the intervention period. As it was not clarified as what point (if ever) the person emerged from PTA it was decided not to include their data in the results.

[19] Only approximations could be made from subtracting the year of injury from the year of publication.

[20] Authors also administered the Aberrant Behaviour Checklist but did not provide any quantitative results for this measure. As such, these results were not included in the review.

					**Other Outcomes**<br>N/A<br><br>**Review Outcomes Not Reported**<br>Primary:<br>Secondary: All - psychological[21] health, cognition, QoL, participation[22]		
**Patterson, 1987**<br><br>**Open trial case series**<br><br>**USA; Inpatient**<br><br>**JBI RoB**<br>**Y: 3**<br>**N: 0**<br>**U: 7**<br>**N/A: 0**	N: 2 (+ 6 ABI patients not included in this review)<br><br>**Sample**<br>- TBI from gunshot wound and hx of agitation, rage, belligerence, resistive and uncooperative behavior, repeated physical attacks (with >1 causing physical harm including fractures to themselves/others)<br><br>Gender<br>Males: 100%<br><br>Age<br>Patient 1: 37yrs<br>Patient 2: 43yrs	**Time post injury**<br>Patient 1: 8yrs<br>Patient 2: 10yrs<br><br>**Severity**<br>NR but EEG findings reported –<br><br>Patient 1: Left frontal slow-wave focus<br>Patient 2: Right temporal slow-wave focus	**Carbamazepine (Anti-convulsant)**<br>Initial<br>Dose: 200mg<br>Freq: 3/day<br><br>Day 2<br>Dose: 200mg<br>Freq: 4/day<br><br>> Day 2<br>200mg 4/day maintained until a carbamazepine level could be obtained (8-12µg/mL; usually 7-10 days to obtain)<br><br>Dur: 2wks (this is the period of data collection; patients did not discontinue treatment at end of study period)	Other:<br>- no participants were on anticonvulsant maintenance therapy	**Primary - Changes in Aggression/Anger**<br>**Assaultive behaviors**<br>All episodes of completed/attempted assaultive behavior were recorded by nursing staff in a log book<br>Duration of observation: 24 hours a day for 7 day intervals<br>Baseline = Two 7 day control periods with a 3 day break period (both control periods were prior to treatment period)<br>Treatment = Two treatment periods with a 3 day break period. First treatment period lasted until carbamazepine level was reached (8-12µg/mL; usually 7-10 days to obtain). Second treatment period was 7 days.<br>Descriptives and paired t-test<br><br>**Primary – Harms**<br>Blood counts, platelet counts, reticulocyte counts<br>Twice weekly<br>‘Routine Laboratory work’<br>As required<br><br>**Other Outcomes**<br>N/A<br><br>**Review Outcomes Not Reported**<br>Primary: Nil<br>Secondary: All - psychological health, cognition, QoL, participation	**Primary - Changes in Aggression/Anger**<br><br>**Assaultive behaviors:**<br>Patient 1<br>- Number of incidents in control periods: 4,3<br>- Number of incidents in treatment periods: 1, 2<br>Patient 2<br>- Number of incidents in control periods: 2, 4<br>- Number of incidents in treatment periods: 1, 1<br>- Anecdotal evidence to suggest that residual episodes occurring after that start of carbamazepine were thought to be less intense and of shorter duration than episodes in the control period.<br>- For entire group (2 TBI, 6 ABI (non-TBI)), there was a statistically significant decline in number of episodes ($p < 0.05$)<br><br>**Harms**<br>- No hematopoietic side effects were observed.<br>- Two patient had transient diplopia and ataxia that cleared spontaneously w/n 1 hr. Note: unclear if these were the 2 TBI patients.	**N: 0**<br>Due to AEs: N/A<br><br>Loss to follow-up: N/A

[23] Findings were only available in a figure with no point estimates provided. As such, these have been approximated from visual inspection of the figures.

[21] The authors did note an improvement in mood for Patient 1, however, this was only provided in narrative text with no quantitative results and so was not included in this review.

[22] The authors did note an improvement in engagement with social activities for Patient 1, however, this was only provided in narrative text with no quantitative results and so was not included in this review.

AE – adverse events; BDI – Beck Depression Inventory; CGI – Clinical Global Impression Scale; DAI – diffuse axonal injury; Dur – Duration; Freq – Frequency; GCS – Glasgow Coma Scale; hx – history; JBI – Joanna Briggs Institute; KAS- Belligerence; Katz Adjustment Scale – Belligerence cluster score; KAS-General Psychopathology - Katz Adjustment Scale – General psychopathology cluster score; LFT – low frustration tolerance; LoC – loss of consciousness; LR – logistic regression; MMSE – Mini Mental Status Examination; MAOI – monoamine oxidase inhibitor; NOS – not otherwise specified; NFI – Neurobehavioral Functioning Inventory; NPI-A - Neuropsychiatric Inventory – Agitation/ Aggression domain; NPI-I – Neuropsychiatric Inventory – Irritability domain; OSSI-I – Organic Signs and Symptoms Inventory – informant response; OSSI-P – Organic Signs and Symptoms Inventory – patient response; POMS – Anger Hostility – Profile of Mood State – Anger Hostility factor score; pts – points; PTA – post traumatic amnesia, RBANS– Repeatable Battery for the Assessment of Neuropsychological Status; RoB – Risk of Bias; SAE – serious adverse event; STAT-S – State-Trait Anger Scale-State; STAS-T – State-Trait Anger Scale – Trait; STAXI-2 – State-Trait Anger Expression Inventory -2 - QoL – quality of life
